# Cascade‐Responsive MXene@Cu‐MOF Heterostructure Integrates Antioxidant Activity, Infection Control, and Vascularization for Tracheal Repair

**DOI:** 10.1002/advs.202521174

**Published:** 2026-01-27

**Authors:** Liang Guo, Yingran Shen, Jiaoyu Yi, Juanjuan Li, Ziming Wang, Erji Gao, Siqiang Zheng, Zhe‐Sheng Chen, Bo Tao

**Affiliations:** ^1^ Department of Thoracic Surgery, Shanghai Pulmonary Hospital, School of Medicine Tongji University Shanghai China; ^2^ Department of Plastic Surgery, Renji Hospital, School of Medicine Shanghai Jiaotong University Shanghai China; ^3^ Department of Medical Oncology, Shanghai Pulmonary Hospital and Thoracic Cancer Institute, School of Medicine Tongji University Shanghai China; ^4^ Department of Pharmaceutical Science, College of Pharmacy and Health Science St John's University Queens New York USA

**Keywords:** cascade‐responsive nanoplatform, heterostructure, multifunction, photothermal effect, tracheal repair

## Abstract

Extensive tracheal repair is limited by oxidative injury, infection, and insufficient vascularization with delayed cartilage maturation. We developed cascade‐responsive MXene@Cu‐MOF heterostructures within gelatin methacryloyl (GelMA) hydrogels to enable staged tracheal repair. Copper‐based metal‐organic framework (Cu‐MOF) nanocrystals grown in situ on ultrathin Ti_3_C_2_T_x_ MXene form an integrated 2D/3D heterointerface, preventing restacking and ensuring uniform “backpack” distribution on a photothermal skeleton. The platform acts in three stages: 1) MXene scavenges radicals, reducing oxidative stress and stabilizing the early environment; 2) Mild acidification triggers pH‐responsive Cu^2+^ release, while near‐infrared light accelerates MOF decomposition to deliver on‐demand Cu^2+^ bursts, enhancing antibacterial efficacy through photothermal heating; 3) Sustained low‐dose Cu^2+^ promotes endothelial proliferation, migration, and tube formation with VEGF, eNOS, HIF‐1α, and FGF2 upregulation, supporting vascular ingrowth. Ring‐to‐tube–fabricated MXene@Cu‐MOF/GelMA tracheal constructs show robust proteoglycans, type II collagen, and biomechanical stiffness. In a rabbit extensive tracheal defect model, MXene@Cu‐MOF/GelMA tracheal grafts improve airway patency and survival, reduce infection and mucus impaction, and enhance epithelial, vascular, and cartilage regeneration. Bulk RNA‐seq confirms suppression of inflammatory pathways and enrichment of antibacterial, angiogenic, and chondrogenic programs. This cascade platform couples photothermal conversion with on‐demand ionic dosing to integrate antioxidant activity, infection control, and vascularization for clinically translatable tracheal repair.

## Graphical abstract

A cascade‐responsive MXene@Cu‐MOF/GelMA hydrogel is engineered as a “skeleton–backpack” platform for extensive tracheal repair. The MXene framework scavenges postoperative ROS and converts NIR light into mild hyperthermia, while the Cu‐MOF component provides pH/NIR‐responsive Cu^2+^ dosing for infection control, angiogenesis, and chondrogenesis. In orthotopic rabbit models, this integrated strategy supports vascularized cartilage regeneration and long‐segment airway reconstruction with favorable systemic safety.

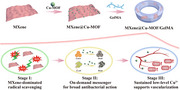

## 1 Introduction

The functional reconstruction of the trachea, a vital conduit of the respiratory system, stands as a largely unsolved frontier in regenerative medicine [1]. Its unique biomechanical and physiological demands, characterized by cartilaginous rigidity and mucociliary clearance, make regeneration following extensive trauma or oncological resection an exceptionally formidable task [2]. The persistent failure of numerous tissue‐engineered trachea (TET) grafts in clinical and preclinical trials points to a sobering reality: our current strategies are fundamentally ill‐equipped to master the hostile and dynamic biological battlefield that emerges at the implant‐host interface [3]. This high failure rate is not merely a technical setback but a critical conceptual bottleneck, revealing a profound disconnect between material design and the complex, orchestrated symphony of natural tracheal healing.

The post‐implantation microenvironment is not a passive recipient of a graft but an active arena where a triad of interconnected pathological events—hyper‐inflammation, bacterial infection, and angiogenic failure—conspire to create a vicious, self‐amplifying cycle of destruction [4]. The initial surgical insult unleashes a “cytokine storm” and an overwhelming burst of reactive oxygen species (ROS), which polarize macrophages toward a pro‐inflammatory M1 phenotype [5]. This aggressive inflammatory milieu, far from being conducive to repair, actively degrades the extracellular matrix (ECM) and creates an immunocompromised, nutrient‐rich landscape—a perfect storm for opportunistic bacterial colonization and subsequent biofilm formation [6]. Once established, these bacterial communities perpetuate a state of chronic, non‐resolving inflammation, effectively locking the system in a destructive feedback loop [7]. This pathophysiological nexus represents the primary roadblock to tracheal regeneration. Within this nexus, essential tracheal healing processes are not just slowed, but actively antagonized. For instance, the very signaling pathways required for angiogenesis, such as those mediated by vascular endothelial growth factor ‌ (VEGF) and hypoxia inducible factor ‐ 1α (HIF‐1α), are profoundly suppressed by pro‐inflammatory cytokines (e.g., tumor necrosis factor‐α (TNF‐α)) and bacterial endotoxins [8, 9]. Without a timely vascular supply, the engineered TET is doomed to ischemic necrosis from its core, transforming the promise of regeneration into a reality of fibrotic scarring and graft failure [10].

This deep‐seated challenge exposes the inherent naivety of conventional biomaterial designs, which often resort to a “functional cocktail” approach—the simplistic physical amalgamation of disparate therapeutic agents [11]. Such strategies are fundamentally flawed because they lack the spatiotemporal intelligence to intervene in the tracheal healing process with the required precision. The passive, uncoordinated release of antibiotics, anti‐inflammatory drugs, and growth factors fails to address the sequential and dynamic nature of the vicious cycle [4]. It is akin to carpet bombing a complex battlefield, causing significant collateral damage—such as inducing drug resistance or suppressing essential immune functions—while failing to hit the right target at the right time [12, 13]. The critical unmet need, therefore, is not for materials with more functions, but for materials with smarter functions. We must transition from creating passive “drug depots” to engineering autonomous “micro‐robotic” systems capable of sensing the local pathological state and executing a logically programmed, multi‐stage therapeutic protocol [14, 15].

To answer this call, we have conceptualized and engineered a paradigm‐shifting solution: a monolithic, intelligent nanoplatform based on a structurally integrated MXene@Cu‐MOF heterostructure. This design represents a deliberate departure from the physical mixture philosophy. By anchoring copper‐based metal‐organic framework (Cu‐MOF) nanocrystals directly onto the 2D MXene nanosheets, we create a single, unified entity where form dictates function, and synergy is hard‐wired into the architecture. We posit that this “all‐in‐one” nanoplatform, when embedded within a hydrogel scaffold, can act as a programmable regenerative conductor, autonomously orchestrating the deconstruction of the pathological vicious cycle and the subsequent initiation of constructive tracheal repair.

**SCHEME 1:**
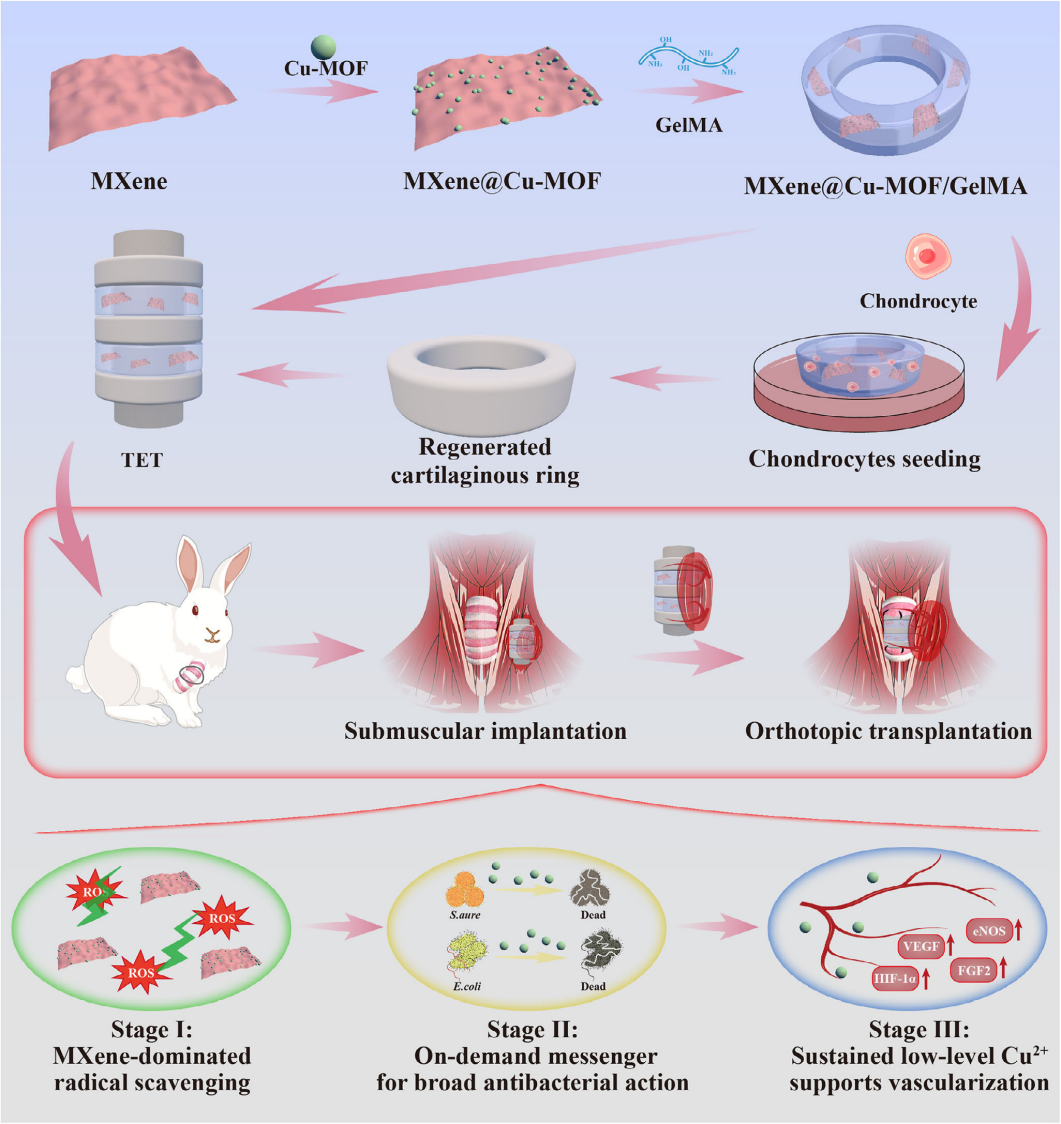
Cascade‐responsive MXene@Cu‐MOF heterostructure integrating antioxidant activity, infection control, and vascularization for tracheal repair. Cu‐MOF nanocrystals are grown in situ on ultrathin Ti_3_C_2_T_x_ MXene to form a unified 2D/3D heterostructure (“skeleton–backpack”) embedded in a GelMA hydrogel. The integrated interface prevents MXene restacking, ensures uniform MOF dispersion, and provides a direct transduction path from photothermal heat to ionic dosing. Stage I: MXene scavenges postoperative ROS and, under mildly acidic conditions, the MOF releases low‐dose Cu^2+^ that stabilizes the early microenvironment. Stage II: brief NIR exposure produces local heating on MXene and accelerates MOF decomposition, yielding an on‐demand Cu^2+^ burst and a photothermal effect that enhances antibacterial effects. Stage III: sustained low‐level Cu^2+^ supports endothelial proliferation, migration, and tube formation (VEGF/eNOS axis), fostering microvessel ingrowth; epithelial continuity and cartilage‐matrix maturation (Col‐II, GAG, stiffness) follow, together contributing to long‐segment tracheal repair.

The therapeutic action of our platform is designed as a pre‐programmed, cascaded biological response. Stage 1: Pre‐emptive environmental conditioning. Immediately upon implantation, the MXene backbone acts as a potent “ROS sponge”, neutralizing the initial inflammatory trigger and preventing the polarization toward a destructive M1 macrophage phenotype. This pre‐emptive immunomodulation is the critical first step to break the cycle before it begins. Stage 2: Adaptive, synergistic host defense. The system then acts as a sentinel, where the acidic pH—a definitive signature of inflammation and infection—triggers the “unlocking” of the Cu‐MOF layer, initiating a controlled release of antibacterial Cu^2+^ ions [16]. This baseline defense can be dramatically escalated on‐demand. In the face of a severe bacterial threat, external near infrared (NIR) irradiation activates MXene's photothermal effect. This localized hyperthermia serves a dual, synergistic purpose: it physically ablates bacteria while simultaneously acting as a secondary key to accelerate the thermo‐sensitive decomposition of the MOFs, unleashing a high‐concentration wave of Cu^2+^ [17]. This “smart bomb” strategy, combining photothermal therapy, offers an exceptionally potent and targeted approach to eradicate even resilient bacteria. Stage 3: Pro‐regenerative pivot. Once the microenvironment is “sanitized” and “pacified”, the accumulated Cu^2+^ ions pivot their biological function, acting as powerful signaling cues to drive angiogenesis and establish the vital vascular network [18]. By designing a platform that first controls inflammation and infection and subsequently promotes vascularization and tissue regeneration, this work introduces a sophisticated, bio‐inspired strategy that moves beyond simple regeneration toward an intelligently orchestrated reconstruction of extensive tracheal defects (Scheme 1).

## 2 Materials and Methods

### 2.1 Materials and Animals

Titanium aluminum carbide (Ti_3_AlC_2_, 400 mesh, >99%) was purchased from Jilin 11 Technology Co., Ltd. (Jilin, China). Lithium fluoride (LiF, 98.5%), hydrochloric acid (HCl, 37 wt.%), copper(II) nitrate trihydrate (Cu(NO_3_)_2_·3H_2_O, 99.5%), and 1,3,5‐benzenetricarboxylic acid (H_3_BTC, 98%) were procured from Sigma–Aldrich (St. Louis, MO, USA). Gelatin methacryloyl (GelMA) was purchased from a commercial supplier (EFL‐GM‐90, Engineering for Life, China). Lithium phenyl‐2,4,6‐trimethylbenzoylphosphinate (LAP, 98%) was obtained from MedChemExpress (Monmouth Junction, NJ, USA). Deionized (DI) water (18.2 MΩ·cm) was used throughout all experiments. All other chemical reagents were of analytical grade and used as received without further purification.

A total of 5 nude mice aged 2 months were obtained from Shanghai SLAC Laboratory Animal Co., Ltd. (Shanghai, China). A total of 20 female New Zealand White rabbits, aged 6 months and weighing approximately 2.5 kg, were obtained from Shanghai Jiagan Biotechnology Co., Ltd. (Shanghai, China). All animal procedures were performed in accordance with institutional guidelines and were approved by the Ethics Committee of Shanghai Pulmonary Hospital (K21‐355Y, Shanghai, China).

### 2.2 Synthesis of Ti_3_C_2_T_x_ (MXene) Nanosheets

Ti_3_C_2_T_x_ nanosheets were prepared by selectively etching the aluminum layer from the MAX phase precursor (Ti_3_AlC_2_) using a minimally intensive lithium fluoride and hydrochloric acid (MILD) method [19]. In a typical procedure, 2.0 g of LiF was dissolved in 40 mL of 9 M HCl in a Teflon beaker under constant magnetic stirring in an ice bath. Subsequently, 2.0 g of Ti_3_AlC_2_ powder was added slowly to the etchant solution over 10 min to avoid an excessive exothermic reaction. The mixture was stirred at 35°C for 24 h. After etching, the resulting suspension was washed repeatedly with DI water by centrifugation (3500 rpm, 5 min per cycle) until the pH of the supernatant reached approximately 6. The sediment was then resuspended in DI water and sonicated for 1 h in an ice bath under a nitrogen atmosphere to facilitate delamination. Finally, the delaminated Ti_3_C_2_T_x_ nanosheet suspension was collected by centrifugation at a higher speed (8000 rpm, 30 min) to remove any un‐etched or large particles. The concentration of the resulting dark green Ti_3_C_2_T_x_ dispersion was determined by weighing the solid content after vacuum drying. The dispersion was stored at 4°C for further use.

### 2.3 In‐Situ Growth of MXene@Cu‐MOF Heterostructures

Cu‐MOF nanocrystals were grown directly on MXene by liquid‐phase in situ synthesis. A Ti_3_C_2_T_x_ dispersion (20 mL, 1 mg/mL) was sonicated for 15 min, then Cu(NO_3_)_2_·3H_2_O (0.5 mmol) was added and stirred for 1 h to adsorb Cu^2+^ onto the surface ─O/─OH terminations. Separately, H_3_BTC (0.33 mmol) was dissolved in 20 mL ethanol/water (1:1, v/v) and added dropwise to the Cu^2+^‐loaded MXene under vigorous stirring. The reaction proceeded at room temperature for 12 h. The product (MXene@Cu‐MOF) was collected by centrifugation (8000 rpm, 10 min), washed three times with ethanol and water, and re‐dispersed in water. Pristine Cu‐MOF (HKUST‐1) was synthesized in parallel without MXene as a control. Unless noted, concentrations of “nanosheets” refer to MXene mass in the heterostructure.

### 2.4 Fabrication of MXene@Cu‐MOF/GelMA Hydrogels

GelMA (10% w/v) was dissolved in phosphate‐buffered saline (PBS) containing LAP (0.5% w/v) at 37°C. MXene@Cu‐MOF was added to yield a final heterostructure concentration of 1 mg/mL unless otherwise specified; mixtures were gently vortexed to ensure homogeneity. Precursor solutions were cast into cylindrical molds (mechanical tests) or custom ring molds (tracheal rings) and photo‐crosslinked under 365 nm UV (10 mW/cm^2^, 10 min) [20]. Matching GelMA hydrogels were prepared as controls.

### 2.5 Physicochemical Characterization

Morphologies of synthesized Cu‐MOF, MXene, and MXene@Cu‐MOF were examined by scanning electron microscopy (SEM; FEI Quanta 250) and transmission electron microscopy (TEM; JEOL JEM‐2100F). Elemental composition and spatial distribution of Cu‐MOF, MXene, and MXene@Cu‐MOF were mapped and calculated by energy dispersive spectroscopy (EDS) coupled to SEM/TEM. Crystal structures of GelMA, Cu‐MOF, MXene, and MXene@Cu‐MOF/GelMA were analyzed by X‐ray diffraction (XRD; Bruker D8 Advance, Cu Kα). Fourier transform infrared spectrometer (FTIR) spectra of Cu‐MOF, MXene, and MXene@Cu‐MOF were collected on a Nicolet iS50 (Thermo Fisher Scientific, USA). The surface elemental composition and chemical states of Cu‐MOF and MXene were further determined by X‐ray photoelectron spectroscopy (XPS; Thermo Scientific Kα+). The hydrodynamic size and ζ‐potential of Cu‐MOF, MXene, and MXene@Cu‐MOF were measured using a Zetasizer Nano ZS instrument (Malvern Panalytical) via dynamic light scattering (DLS). Atomic force microscopy (AFM; Bruker, USA) was used for thickness/topography of Cu‐MOF, MXene, and MXene@Cu‐MOF.

### 2.6 Photothermal Performance and Stability

Aqueous dispersions of MXene@Cu‐MOF (typically 1.0 mg/mL) and GelMA and MXene@Cu‐MOF/GelMA hydrogels (normalized to 1.0 mg/mL nanosheets) were irradiated with an 808 nm NIR laser at the indicated power densities (0.5–1.5 W/cm^2^). PBS served as the control group. Temperature–time curves were recorded by a thermocouple and/or infrared thermal camera. In addition, temperature–concentration (0.5–1.5 mg/mL) curves at fixed power densities (1.5 W/cm^2^) for MXene@Cu‐MOF/GelMA were also investigated. For cycling stability, samples underwent repeated on/off irradiation (10 min on, 10 min off; up to 10 cycles).

### 2.7 pH‐, NIR‐, and Thermal‐Responsive Cu^2+^ Release

MXene@Cu‐MOF (dispersion or GelMA hydrogel) was incubated in buffers at pH varying from 5.0 to 7.8 at 37°C and 45°C. At preset times, samples were centrifuged and supernatants analyzed for Cu^2+^ by inductively coupled plasma mass spectrometry (ICP‐MS) against calibration standards. For NIR‐triggered release, samples (pH 6.5) received 808 nm irradiation (1.0 W/cm^2^, 10 min) at specified intervals before sampling. Cumulative release was reported as a function of time, pH, temperature, and irradiation.

In the NIR‐triggered Cu^2+^ release experiments, MXene@Cu‐MOF/GelMA hydrogel discs were immersed in PBS at 37°C. Samples were not exposed to continuous laser irradiation over the entire 0–25 h period. Instead, an intermittent “on/off” schedule was employed: for the +NIR groups, the hydrogels were irradiated with an 808 nm laser at 1.0 W/cm^2^ for 10 min at each scheduled sampling time point, and kept in the dark for the remaining time between pulses. Immediately after each irradiation, aliquots of the supernatant were collected for Cu^2+^ quantification, while the ‐NIR groups were maintained in the dark throughout under otherwise identical conditions. After the final irradiation, all samples were further incubated without NIR exposure until the end of the 25 h observation period.

The cumulative Cu^2+^ release from MXene@Cu‐MOF/GelMA hydrogels was evaluated in PBS at pH 7.4 and 6.8. Hydrogels containing MXene@Cu‐MOF were immersed in PBS (pH 7.4 or 6.8) and incubated at 37°C under gentle shaking. At predetermined time points, the supernatant was collected and the Cu^2+^ concentration was quantified by ICP‐MS.

Furthermore, Cu^2+^ release from MXene@Cu‐MOF/GelMA hydrogels in PBS at pH 7.4 and 6.8 was examined with and without NIR irradiation. For the +NIR groups, samples were irradiated with an 808 nm laser (1.0 W/cm^2^, 10 min) prior to sampling at each specified time point, while the ‐NIR groups were kept in the dark under otherwise identical conditions. The collected supernatants were analyzed for Cu^2+^ by ICP‐MS.

### 2.8 In Vitro Biocompatibility Evaluation

Cytocompatibility was evaluated by seeding primary chondrocytes onto GelMA, MXene/GelMA, and MXene@Cu‐MOF/GelMA hydrogels and assessing cell metabolic activity (CCK‐8, Sigma, USA), viability (Calcein‐AM/PI Live/Dead staining, MCE, USA), and morphology/spreading (F‐actin cytoskeleton and 4',6‐diamidino‐2‐phenylindole (DAPI) nuclear staining) at 1, 4, and 7 days post‐treatment. Primary chondrocytes without any treatment served as the blank control group.

### 2.9 Radical‐Scavenging Assays (cell‐free)

2,2'‐Azinobis(3‐ethylbenzothiazoline‐6‐sulfonic acid) radical cation (ABTS•^+^) and 2,2‐Diphenyl‐1‐picrylhydrazyl radical (DPPH•) scavenging: MXene@Cu‐MOF (0–1.0 mg/mL) was mixed with ABTS•^+^ or DPPH• working solutions; after 30 min in the dark, absorbance decreases at 734 nm (ABTS•^+^) and 517 nm (DPPH•) were recorded and scavenging ratios calculated. Superoxide (O_2_•^−^) and hydroxyl (•OH) scavenging were quantified by UV–vis readouts of standard probe reactions, following the same concentration range and timing used for ABTS/DPPH.

### 2.10 Cellular ROS Assays

RAW264.7 macrophages (ATCC, Manassas, VA, USA) were challenged with H_2_O_2_ or lipopolysaccharide (LPS; Macklin Biochemical Co., Ltd. China) and then treated with PBS (blank), GelMA extract, MXene/GelMA extract, or MXene@Cu‐MOF/GelMA extract. Intracellular ROS levels were stained with 2',7'‐Dichlorodihydrofluorescein diacetate (DCFH‐DA) and imaged by fluorescence microscopy; parallel samples were analyzed by flow cytometry. Extraction was performed by incubating hydrogels in complete medium (surface area/volume ≈ 3 cm^2^/mL) at 37°C for 24 h.

### 2.11 In Vitro Antibacterial Assays

Escherichia coli (E. *coli*) and Staphylococcus aureus (S. *aureus*) were used as model Gram‐negative and Gram‐positive bacteria. In the co‐incubation assay, bacterial suspensions were exposed to near‐infrared (808 nm, 1.0 W/cm^2^, 10 min) and then treated with PBS (blank), pure GelMA, MXene@Cu‐MOF, or MXene@Cu‐MOF/GelMA for 24 h. At the end of the treatment, the bacterial suspensions were immediately collected, serially diluted, and spread on nutrient agar plates, which were incubated at 37°C for 24 h. The bacterial coverage area (%) was then quantified from the resulting colonies. Fluorescence‐based viability imaging was performed using the Live/Dead BacLight bacterial viability kit (Thermo Fisher, USA). After NIR treatment, the samples were gently rinsed with PBS, and bacteria remaining on and around the material surface were stained with the SYTO9/PI reagents according to the manufacturer's instructions. Fluorescence images were acquired within 15 min after staining without further incubation. Representative post‐treatment bacterial morphologies were additionally examined by SEM.

### 2.12 Anti‐Inflammatory Polarization of Macrophages

RAW264.7 cells were pre‐stimulated with LPS (positive inflammatory control), then co‐cultured with hydrogel extracts (including pure GelMA, MXene/GelMA, and MXene@Cu‐MOF/GelMA groups) as above. RAW264.7 cells treated with interleukin‐4 (IL‐4) were set as a negative control. Immunofluorescence staining for CD86 (M1) and CD206 (M2) and Western blots for inducible nitric oxide synthase (iNOS), interleukin‐6 (IL‐6), TNF‐α, and interleukin‐1β (IL‐1β) were performed using standard protocols.

### 2.13 In Vitro Angiogenesis Assay under NIR Irradiation

Human umbilical vein endothelial cells (HUVECs; ATCC, Manassas, VA, USA) were used. All angiogenesis assays in this section were conducted under NIR irradiation, consistent with the optothermal settings used elsewhere in this study (808 nm, 1.5 W/cm^2^; single 10 min exposure unless otherwise indicated). Experimental groups were: Blank (PBS), GelMA, MXene@Cu‐MOF, and MXene@Cu‐MOF/GelMA. For extract‐based conditions, cells were incubated with material extracts prepared in complete endothelial growth medium; for hydrogel conditions, extracts were used throughout the assay period. (1) EdU incorporation. HUVECs were seeded on glass‐bottom plates, allowed to attach, then treated with the indicated extracts. After NIR exposure, cells were pulsed with EdU following the manufacturer's instructions. Nuclei were counterstained with DAPI. Percent EdU^+^ nuclei was quantified from ≥5 random fields per well using ImageJ; values were averaged per well and per experiment. (2) Scratch (wound‐closure) migration. Confluent HUVEC monolayers were scratched with a sterile 200 µL tip to create a linear wound, rinsed to remove debris, overlaid with the indicated extracts, and irradiated with NIR as above. Phase‐contrast images at 0 h and 24 h were analyzed in ImageJ to calculate wound closure (%) = [1 − (wound area_t / wound area_0)] × 100. (3) Tube formation. Growth factor–reduced Matrigel (pre‐chilled) was added to 96‐well plates and gelled at 37°C. HUVECs were seeded onto Matrigel in medium containing the indicated extracts and irradiated with NIR as above. After incubation (per figure legend), phase‐contrast or fluorescence images were analyzed with Angiogenesis Analyzer (ImageJ) to quantify branch points and total tube length. (4) RT‐qPCR. Total RNA was isolated from HUVECs using a TRIzol method and reverse‐transcribed to cDNA with a commercial kit following the manufacturer's protocol. Quantitative PCR was performed using SYBR Green chemistry on a real‐time instrument with cycling conditions typical for amplicons of 80–200 bp (e.g., 95°C 10 min; 40 cycles of 95°C 15 s, 60°C 60 s), followed by melt‐curve analysis to confirm specificity. Target transcripts included *VEGF*, endothelial nitric oxide synthase (*eNOS*), *HIF‐1α*, and fibroblast growth factor‐2 (*FGF2*). Expression was normalized to the GAPDH housekeeping gene and analyzed by the 2^−ΔΔCt^ method. Primer sequences and amplicon sizes are provided in Table S1.

### 2.14 Fabrication of TET Rings, In Vitro Maturation, and Subcutaneous Implantation

Cartilaginous rings were fabricated using molds incorporating the MXene@Cu‐MOF/GelMA platform as indicated [21]. Rings were cultured up to 4 weeks in chondrogenic medium; histology (hematoxylin and eosin (HE), Safranin‐O) and immunohistochemistry (type II collagen, Col‐II) were performed on fixed sections.

Thereafter, the in vitro engineered cartilaginous rings and ring‐shaped MXene@Cu‐MOF/GelMA scaffolds were alternatively stacked onto a silicone tube to form a composite construct using a previously established method [21]. Constructs were implanted subcutaneously in nude mice for 4 weeks. Animals were anesthetized, dorsal pockets were created, and sterilized constructs were inserted. Explants were fixed and analyzed by HE, Safranin‐O, Toluidine Blue, and immunohistochemistry Col‐II; vascular structures were annotated in representative fields. Glycosaminoglycan (GAG) content was quantified by 1,9‐Dimethylmethylene Blue assay; Col‐II content by enzyme‐linked immunosorbent assay (R&D Systems, Minneapolis, MN, USA); compressive Young's modulus was measured on cylindrical samples at a fixed strain rate. Native tracheal tissue from New Zealand White rabbits was used as the control group.

### 2.15 Orthotopic Repair of Extensive Tracheal Defects in Rabbits

A long‐segment tracheal defect model was established in rabbits. After anesthesia and sterile preparation, an extensive segment was resected and repaired with an MXene@Cu‐MOF/GelMA engineered TET (EXP, n = 10) or a pure GelMA engineered TET (CON, n = 10). Anastomoses were completed per standard technique. During the rabbit orthotopic tracheal repair study, NIR was delivered on an as‐needed basis—triggered when bronchoscopy or clinical monitoring indicated elevated infection/inflammation risk—under brief anesthesia via percutaneous 808 nm irradiation (≈1.5 W/cm^2^, 10 min per session), with real‐time infrared thermometry to maintain the graft‐surface temperature within the mild‐hyperthermia window (≈ 45–50°C).

Postoperative assessments included bronchoscopy at 4 weeks, survival monitoring up to 8 weeks (Kaplan–Meier), and endpoint tissue collection (2–4 weeks) for quantitative metrics (body weight change, tracheal patency ratio, mucous impaction score, GAG/Col‐II content, compressive modulus). Histology (HE, Safranin‐O) and immunohistochemistry staining for Col‐II, CD31 (angiogenesis), cytokeratin (epithelialization), CD68 (macrophages), and CD3 (T cells) were performed. Bacterial burden was assessed by fluorescence in situ hybridization (FISH) and tissue ROS by oxidative‐stress staining. Western blot and RT‐qPCR analyses of the chondrogenic markers (Aggrecan and Col‐II) in the regenerated tracheal tissues were also performed.

At 1 and 4 weeks post‐surgery, animals were euthanized, and blood (for serum) and major organs, including the heart, liver, spleen, lung, and kidney, were collected. Tissue samples were rinsed with physiological saline to remove residual blood, gently blotted dry, weighed, and digested with concentrated HNO_3_ at 60–70°C until complete dissolution, followed by dilution to an appropriate volume with ultrapure water. The Cu content in each sample was quantified by ICP‐MS. Serum samples were diluted with 1% (v/v) HNO_3_ and directly analyzed by ICP‐MS. The number of animals was n = 3 per group per time point.

### 2.16 RNA‐seq and Bioinformatic Analysis

Total RNA from repaired tracheal tissues (CON vs EXP) at 2 weeks was extracted, quality‐checked, and used for library preparation and high‐throughput sequencing. Clean reads were aligned to the reference genome; differential expression analysis (adjusted *p* < 0.05; |log_2_FC| ≥ 1) identified differentially expressed genes (DEGs). Gene ontology (GO) and kyoto encyclopedia of genes and genomes (KEGG) enrichment were computed on upregulated genes; Gene set enrichment analysis (GSEA) was performed on curated gene sets relevant to antibacterial defense, inflammatory response, angiogenesis, and chondrogenesis. Principal component analysis (PCA), volcano plots, and heatmaps were generated to summarize global and sample‐wise differences.

### 2.17 Statistical Analysis

Data are presented as mean ± SD from ≥3 independent experiments unless indicated. Group comparisons used two‐tailed Student's t‐tests (two groups) or one‐way ANOVA with Tukey's post hoc test (≥3 groups). Significance thresholds: *p* < 0.05, *p* < 0.01, *p* < 0.001. For experiments with explicit factorial designs (e.g., ±NIR across materials), two‐way ANOVA was used where appropriate. Survival differences were analyzed by the log‐rank test.

## 3 Results and Discussion

### 3.1 Structural Integration and Physicochemical Signatures of the MXene@Cu‐MOF Heterostructure

**FIGURE 1:**
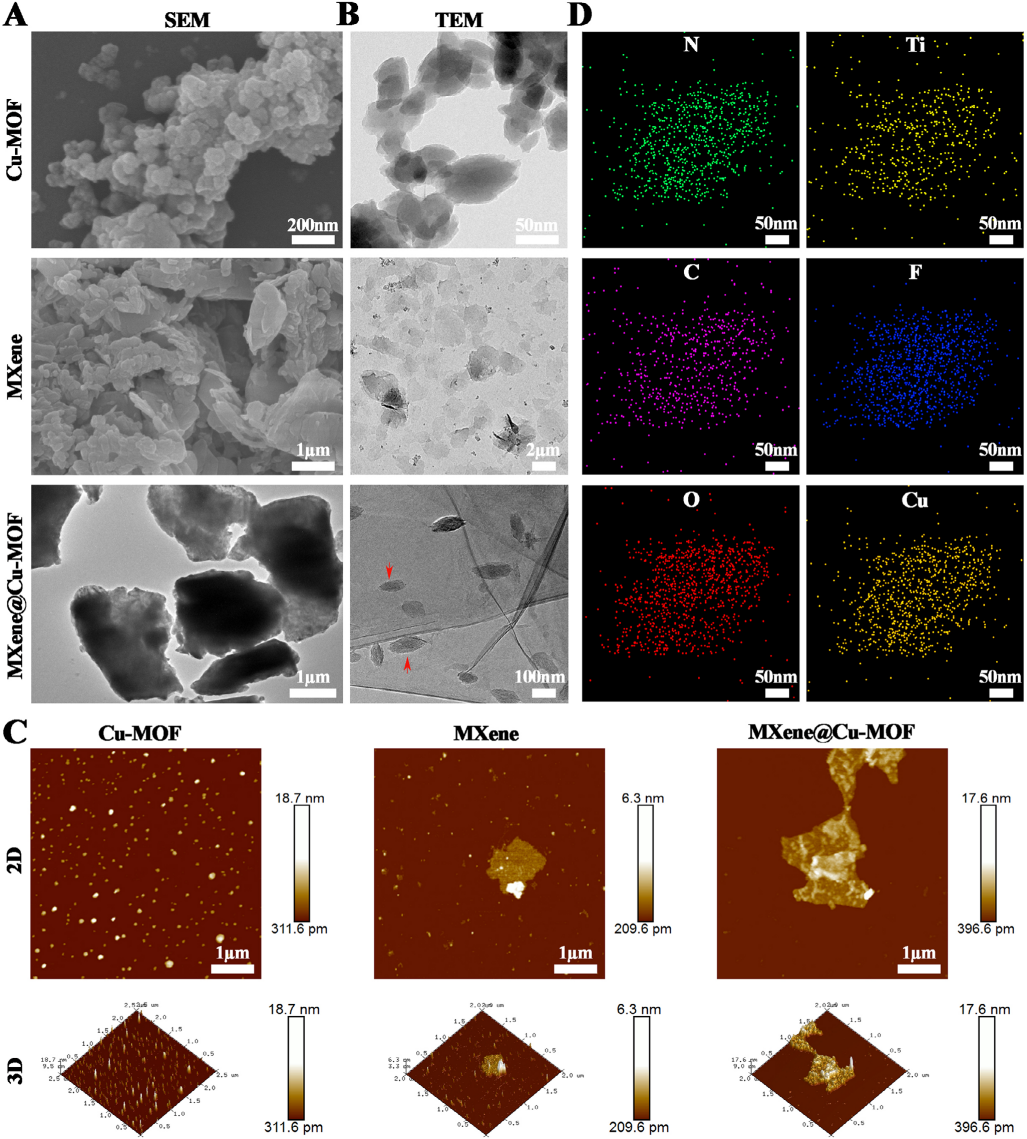
Structural and morphological characterization of the synthesized MXene@Cu‐MOF nanoplatform. A) SEM micrographs of Cu‐MOF, MXene (Ti_3_C_2_T_x_), and the MXene@Cu‐MOF heterostructure. B) TEM micrographs of Cu‐MOF, MXene, and MXene@Cu‐MOF, showing MOF nanocrystals anchored on MXene nanosheets (red arrows indicate representative domains). C) AFM topography of Cu‐MOF, MXene, and MXene@Cu‐MOF, including 2D height maps with corresponding 3D surface renderings. D) EDS elemental maps of the synthesized MXene@Cu‐MOF, showing the spatial distribution of N, Ti, C, F, O, and Cu.

SEM and TEM show that Cu‐MOF nanocrystals are predominantly associated with ultrathin Ti_3_C_2_T_x_ (MXene) nanosheets (Figure 1A,B). The MXene presents as high‐aspect‐ratio sheets with lateral dimensions from hundreds of nanometers to micrometers and a few‐nanometer thickness, with locally crumpled basal planes and clean edges typical of delaminated Ti_3_C_2_T_x_. Three observations indicate that Cu‐MOF crystals are anchored to MXene rather than simply co‐present: edge‐preferential decoration with punctate coverage on the basal plane and few free Cu‐MOF particles in the background; intimate contact without visible interparticle gaps at the MOF–MXene interface; and increased surface roughness with nanoscale height puncta/features on AFM that match Cu‐MOF domains superimposed on a relatively flat MXene baseline (Figure 1C). DLS data of standalone Cu‐MOF dispersions display a narrow, unimodal distribution in the 40–100 nm range with a modest polydispersity index (Figure S1), consistent with the discrete domains seen by electron microscopy.

Elemental maps of the synthesized MXene@Cu‐MOF further support co‐localized N, Ti, C, F, O and Cu signals within the heterostructure (Figure 1D). Stand‐alone controls corroborate component identities: Cu‐MOF shows N, Cu and O, with C from the organic linker (Figure S2), whereas MXene shows Ti and C with O/F signals (Figure S3).

EDS confirmed the expected element sets for each component and their combination (Table S2). For Cu‐MOF, the spectrum contained C, N, O, Cu with the following bulk composition (normalized): C 40.43 wt.% (57.14 at%), N 5.89 wt.% (7.14 at%), O 26.93 wt.% (28.57 at%), Cu 26.74 wt.% (7.14 at%). For MXene, the signal was dominated by Ti, accompanied by C, O, F (no N or Cu): Ti 71.08 wt.% (42.86 at%), C 11.89 wt.% (28.57 at%), O 9.50 wt.% (17.14 at%), F 7.52 wt.% (11.43 at%). The MXene@Cu‐MOF sample exhibited contributions from both phases—C, N, O, Cu, Ti, F—with intermediate levels: Ti 49.76 wt.% (24.79 at%), Cu 8.02 wt.% (3.01 at%), C 20.45 wt.% (40.61 at%), O 14.73 wt.% (21.96 at%), N 1.77 wt.% (3.01 at%), F 5.27 wt.% (6.61 at%). Overall, the composition profile corroborates successful construction of the MXene@Cu‐MOF heterostructure.

Vibrational and surface‐chemical analyses confirm component identity and interface formation. In FTIR, MXene shows bands near 1618 cm^−1^ (C═O) and 3425 cm^−1^ (O─H), consistent with ─O/─OH terminations [22, 23]; the Cu‐MOF displays bands at 1015 cm^−1^ (C─N) assignable to ring vibrations, 1375 cm^−1^ (C─O) assignable to carboxylate‐related vibrations, and 1605 cm^−1^ assignable to aromatic C═C [24]. In MXene@Cu‐MOF, MXene and Cu‐MOF signatures coexist with moderate peak shifts and attenuations, consistent with coordination to Cu nodes and hydrogen‐bonding or electrostatic interactions at the MXene surface (Figure S4). XPS wide scans detect Cu from the MOF and Ti, C, and O from MXene (Figure S5A,B). The Cu 2p region in MOF‐containing samples exhibits the Cu 2p_2_/_3_ main line with shake‐up satellites characteristic of Cu(II), supporting the expected coordination environment. The Ti 2p region of pristine MXene shows components assignable to Ti─C and Ti─O, indicating coexisting carbide backbones and terminated species.

Consistent with these results, XRD further confirms successful integration of the three components (Figure S6). Pristine MXene displays a strong (002) low‐angle reflection at ∼6–7° together with several weaker high‐angle reflections, while Cu‐MOF exhibits multiple sharp “fingerprint” peaks in the 10–40° range, and GelMA shows only a broad amorphous halo centered around ∼20°. In the MXene@Cu‐MOF/GelMA composite, the characteristic MXene (002) peak is still discernible at low angle, and several representative Cu‐MOF reflections remain visible in the 10–40° region, although both sets of peaks show reduced intensity and moderate broadening and are superimposed on the GelMA amorphous background. This pattern indicates that key features of the MXene lattice and Cu‐MOF crystalline domains are largely retained after assembly, while the attenuation and broadening are consistent with the formation of nanoscale MOF crystallites anchored on partially restacked MXene sheets and embedded within the hydrogel matrix.

Electrokinetic measurements are consistent with uniform surface coverage. Both Cu‐MOF and MXene are net‐negative under the tested electrolyte; the composite shows an intermediate negative zeta potential (Figure S7; mean ± SD, n = 3). This averaging implies that the outer slip plane is governed by the composite interface rather than either component alone, in line with conformal MOF deposition on MXene [25].

Taken together, microscopy, elemental mapping, AFM, FTIR, XPS, XRD, and zeta potential measurements establish a structure‐integrated heterojunction rather than a physical mixture. This structural integration is central to the innovation of this work. Oxygen‐terminated Ti_3_C_2_T_x_ provides Cu^2+^ anchoring and heterogeneous nucleation sites, enabling selective in situ Cu‐MOF growth on basal planes and edges under mild liquid‐phase conditions—an approach that is synthetically feasible and scalable [26]. In the resulting architecture, Cu‐MOF grains act as nanoscale spacers that suppress MXene restacking and enforce uniform dispersion, thereby maximizing accessible surface area and interfacial contact.

### 3.2 Broad‐Spectrum ROS Scavenging in Cell‐Free and Cellular Settings

**FIGURE 2:**
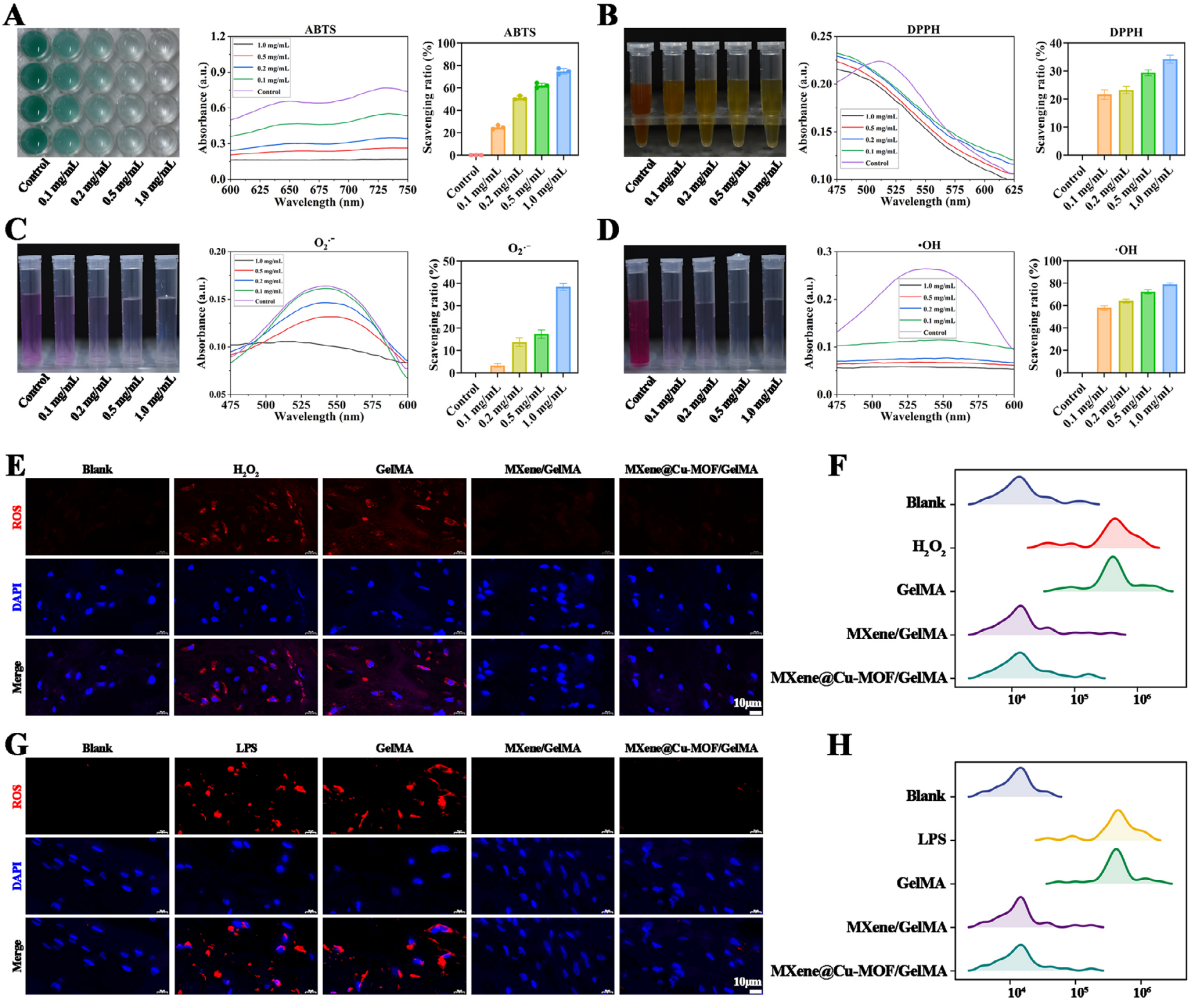
In vitro antioxidant activity of the synthesized MXene@Cu‐MOF nanoplatform. A) UV–vis spectra and corresponding quantification of ABTS•^+^ scavenging after treatment with MXene@Cu‐MOF at 0, 0.1, 0.2, 0.5, and 1.0 mg/mL. B) UV–vis spectra and quantification of DPPH• scavenging by MXene@Cu‐MOF at 0, 0.1, 0.2, 0.5, and 1.0 mg/mL. C) UV–vis spectra and quantification of superoxide (O_2_•^−^) scavenging by MXene@Cu‐MOF at 0, 0.1, 0.2, 0.5, and 1.0 mg/mL. D) Hydroxyl radical (•OH) scavenging by MXene@Cu‐MOF at 0, 0.1, 0.2, 0.5, and 1.0 mg/mL, evaluated by UV–vis (representative spectra and corresponding quantitative analysis). E) Representative fluorescence micrographs of intracellular ROS in RAW264.7 macrophages stained with DCFH‐DA (red) under the following treatments: PBS (blank), H_2_O_2_ only, H_2_O_2_ + GelMA, H_2_O_2_ + MXene/GelMA, and H_2_O_2_ + MXene@Cu‐MOF/GelMA. F) Flow‐cytometric quantification of cellular ROS (DCF‐based) fluorescence for the groups in (E). G) Representative DCFH‐DA staining (red) of intracellular ROS in RAW264.7 macrophages treated with: PBS (blank), LPS alone, LPS + GelMA, LPS + MXene/GelMA, and LPS + MXene@Cu‐MOF/GelMA. H) Flow‐cytometric quantification of cellular ROS (DCF‐based) fluorescence for the groups in (G). Data are presented as mean ± SD (n = 3).

In cell‐free systems, MXene@Cu‐MOF heterostructure displayed a clear concentration‐dependent antioxidant effect across all probe systems. In ABTS and DPPH assays, increasing the material concentration from 0 to 0.1, 0.2, 0.5, and 1.0 mg/mL produced progressively larger reductions in radical absorbance, with stepwise increases in calculated scavenging ratios (Figure 2A,B). O_2_•^−^ and •OH assays showed the same monotonic trend (Figure 2C,D), as evidenced by consistent decreases in the diagnostic chromogenic signals and supporting statistics. These results indicate that antioxidant capacity scales with MXene@Cu‐MOF concentration within the tested range.

Early after implantation, tissues experience (i) a reactive‐oxygen burst and (ii) innate immune activation. We therefore used two canonical stimuli in RAW264.7 macrophages to emulate these pressures: H_2_O_2_ to model oxidative stress and LPS (TLR4 agonist) to model endotoxin‐driven inflammatory activation. This pairing enabled us to test whether the materials suppress intracellular ROS across chemically induced (H_2_O_2_) and receptor‐mediated (LPS) contexts.

In H_2_O_2_–challenged RAW264.7 cells, DCFH‐DA imaging showed near‐baseline intracellular ROS in the PBS (Blank), MXene/GelMA, and MXene@Cu‐MOF/GelMA groups, whereas the H_2_O_2_ and H_2_O_2_ + GelMA groups remained strongly ROS‐positive, indicating that GelMA alone is redox‐inert under our conditions (Figure 2E; Figure S8A). Flow cytometry corroborated these findings: intracellular ROS was lowest with MXene@Cu‐MOF/GelMA, comparable between MXene/GelMA and Blank, and markedly higher in H_2_O_2_ and H_2_O_2_ + GelMA (Figure 2F). The same hierarchy was observed in LPS‐stimulated macrophages, where Blank, MXene/GelMA, and MXene@Cu‐MOF/GelMA maintained low ROS, while LPS and LPS + GelMA remained high (Figure 2G,H; Figure S8B).

The broad scavenging response arises primarily from the redox‐active Ti_3_C_2_T_x_ surface. Electron‐rich basal planes and oxygen‐containing terminations support electron or proton transfer to multiple radical species, explaining the parallel improvements across assays [27, 28]. The heterostructure further strengthens this behavior in two practical ways. First, Cu‐MOF nanocrystals act as nanoscale spacers that limit MXene restacking, maintain accessible surface area, and expose reactive terminations [29, 30], consistent with the concentration‐dependent effects in Figure 2A–D. Second, GelMA encapsulation preserves activity: the hydrogel mesh allows rapid diffusion of small oxidants and probe molecules to the material surface, while immobilization reduces secondary aggregation and nonspecific fouling [31]. Consequently, MXene/GelMA already drives intracellular ROS toward baseline, and MXene@Cu‐MOF/GelMA performs at least as well or better. Functionally, this early suppression of oxidative stress stabilizes the local microenvironment and establishes the first stage of the platform's cascade logic. This is consistent with reports that controlling oxidative and inflammatory cues improves graft integration and regeneration in airway and tissue‐engineering settings, and it aligns with recent work showing that immuno‐supportive scaffolds enhance repair by modulating oxidative stress and macrophage responses [32, 33].

### 3.3 Anti‐Inflammatory Polarization in Macrophages

Because early post‐implantation microenvironments are rich in ROS and pro‐inflammatory cues, macrophage fate (M1‐like vs M2‐like) is a key determinant of downstream healing. We therefore asked whether the MXene‐based platforms could reprogram macrophage polarization toward a pro‐resolution state. RAW264.7 cells were chosen as a well‐established murine macrophage model, and LPS prestimulation was used to generate a robust M1‐biased phenotype via TLR4–NF‐κB/iNOS signaling—mimicking infection‐ or surgery‐associated inflammation. To provide a reference for alternative activation, an IL‐4 group was included as a pro‐M2 benchmark control, alongside GelMA controls.

In LPS‐prestimulated RAW264.7 macrophages, MXene/GelMA and MXene@Cu‐MOF/GelMA both induced a clear shift toward an M2‐like phenotype. Immunofluorescence showed reduced CD86 (M1 marker) and increased CD206 (M2 marker) staining relative to LPS and LPS+GelMA (Figure S9A,B). Quantification of %‐positive cells confirmed lower CD86 and higher CD206 in both MXene/GelMA and MXene@Cu‐MOF/GelMA groups (*p* < 0.001 vs LPS; n = 3). Consistently, Western blots demonstrated reduced iNOS and decreased expression of pro‐inflammatory cytokines (IL‐6, TNF‐α, IL‐1β) in both MXene/GelMA and MXene@Cu‐MOF/GelMA relative to LPS and LPS+GelMA (Figure S9C,D; *p* < 0.001; n = 3). These data indicate that the MXene@Cu‐MOF/GelMA heterostructure (and MXene/GelMA) attenuates inflammatory signaling and bias macrophages toward a pro‐resolution (M2‐like) state under a stringent LPS challenge.

The polarization shift reflects convergent, material‐encoded cues that operate on complementary timescales and are intrinsic to the heterostructure design [34]. The MXene backbone rapidly lowers oxidative tone by quenching extracellular and intracellular ROS (Section 3.2), which is expected to reduce activation of redox‐sensitive inflammatory pathways and the iNOS–NO axis [35]. The near‐baseline DCF signals observed for MXene/GelMA and MXene@Cu‐MOF/GelMA in both H_2_O_2_ and LPS models (Figure 2E–H; Figure S8) are consistent with this interpretation and align with reports that redox normalization facilitates a shift away from M1‐dominant programs in macrophages [36]. GelMA provides a permissive, immobilizing environment that preserves bioactivity while limiting secondary aggregation [37]. Although GelMA itself does not scavenge ROS under our conditions (Figure 2E–H), its mesh permits rapid diffusion of small oxidants to MXene surfaces, while its adhesive motifs and compliant mechanics are compatible with M2‐favoring cell–matrix interactions reported for immuno‐supportive scaffolds [38].

### 3.4 Photothermal Performance and NIR‐Accelerated Cu^2+^ Release

**FIGURE 3:**
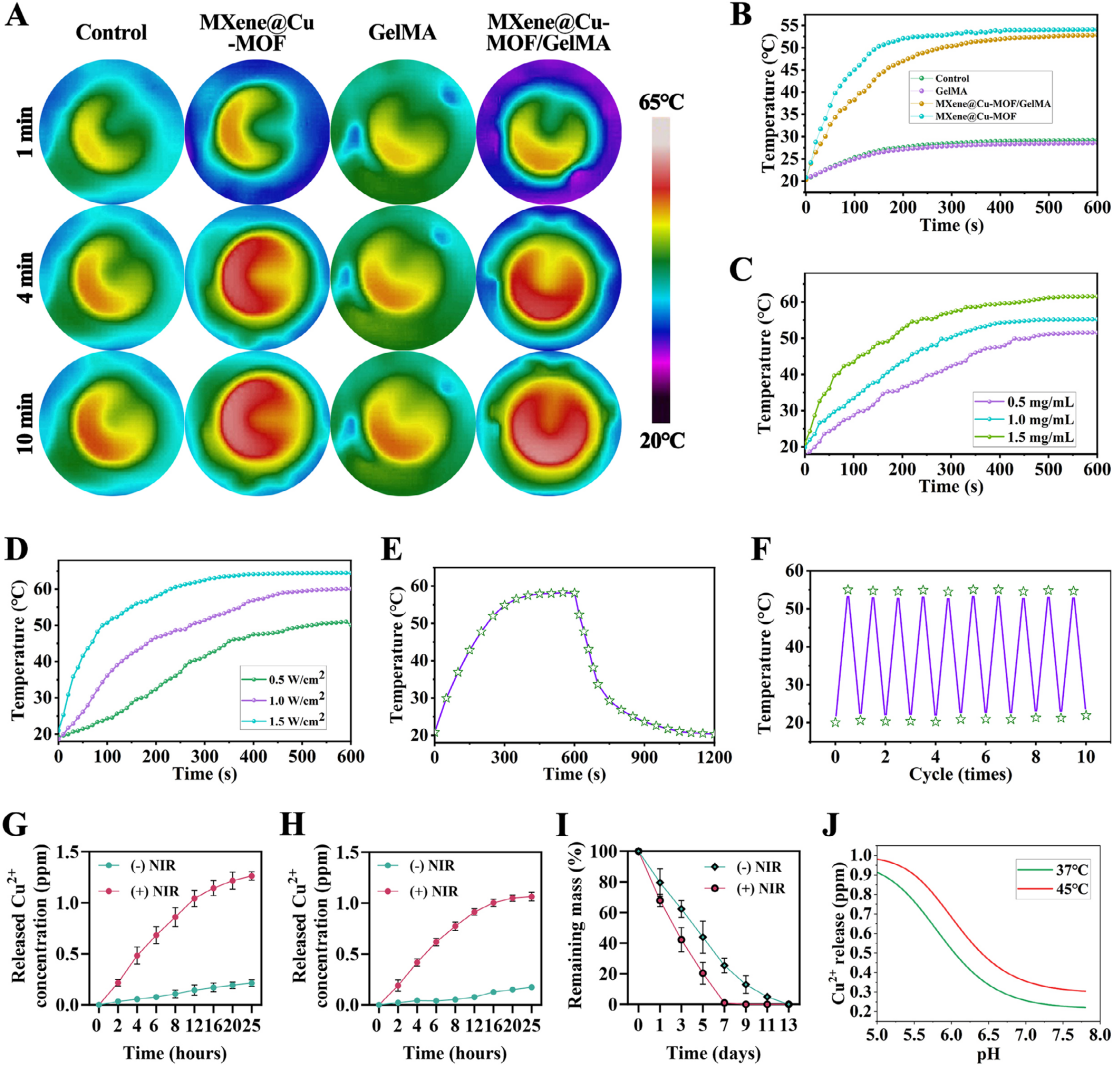
Photothermal behavior of the MXene@Cu‐MOF/GelMA platform. A) Infrared thermal images of Control (PBS), MXene@Cu‐MOF, GelMA, and MXene@Cu‐MOF/GelMA (all at 1.0 mg/mL with respect to MXene nanosheets) under NIR irradiation (1.5 W/cm^2^, 10 min). B) Corresponding temperature–time profiles for the groups in (A). C) Temperature rise of MXene@Cu‐MOF/GelMA at different nanosheet concentrations (0.5, 1.0, and 1.5 mg/mL) under NIR irradiation (1.5 W/cm^2^). D) Temperature rise of MXene@Cu‐MOF/GelMA at a fixed concentration (1.0 mg/mL) under different irradiances (0.5, 1.0, and 1.5 W/cm^2^). E) Heating–cooling curve of MXene@Cu‐MOF/GelMA (1.0 mg/mL) during 10 min NIR irradiation followed by 10 min natural cooling (1.5 W/cm^2^). F) Photothermal stability of MXene@Cu‐MOF/GelMA: 10 repeated heating–cooling cycles at 1.0 mg/mL under NIR irradiation (1.5 W/cm^2^) per cycle. G) In vitro Cu^2+^ release kinetics from MXene@Cu‐MOF with or without NIR irradiation. H) In vitro Cu^2+^ release kinetics from MXene@Cu‐MOF/GelMA with or without NIR irradiation. I) In vitro degradation profiles of MXene@Cu‐MOF/GelMA in PBS (pH 7.4), with or without NIR irradiation. J) pH‐dependent Cu^2+^ release from Cu‐MOF archetypes and the effect of NIR heating. Data are presented as mean ± SD (n = 3).

Under 1.5 W/cm^2^ NIR irradiation, MXene@Cu‐MOF/GelMA exhibited a rapid temperature rise that exceeded PBS and GelMA and approximately mirrored the behavior of MXene@Cu‐MOF dispersions at an equivalent nanosheet dose (1.0 mg/mL) (Figure 3A,B). MXene (Ti_3_C_2_T_x_) is a highly efficient light‐to‐heat converter and supports robust photothermal heating in hydrogels [39]. The temperature increase scaled monotonically with heterostructure concentration from 0.5 to 1.5 mg/mL (Figure 3C) and with irradiance from 0.5 to 1.5 W/cm^2^ (Figure 3D). Heating–cooling curves were reversible over repeated on/off cycles with minimal loss of performance (Figure 3E,F), indicating good photothermal stability of the heterostructure within the hydrogel [40]. Using standard energy‐balance analysis of the heating/cooling traces, the MXene@Cu‐MOF dispersion yielded an efficiency of ∼30.4% at 808 nm and 1.5 W/cm^2^. The MXene@Cu‐MOF/GelMA hydrogel showed a comparable η of ∼29.3% under identical conditions, indicating that GelMA encapsulation preserves light‐to‐heat conversion while slightly altering heat transfer and steady‐state temperature due to matrix‐mediated conduction/convection.

Under intermittent NIR irradiation, Cu^2+^ release from both MXene@Cu‐MOF dispersions and MXene@Cu‐MOF/GelMA hydrogels was markedly accelerated compared with the corresponding dark controls over 0–25 h (Figure 3G,H). The cumulative release curves exhibited stepwise increases coinciding with each irradiation cycle, indicating that short, intermittent NIR pulses can provide an on‐demand enhancement of Cu^2+^ liberation superimposed on the baseline pH‐ and time‐dependent release. This behavior is consistent with previous reports that NIR/thermal inputs can promote partial MOF framework degradation and thereby trigger or accelerate cargo/ion release [41]. In parallel, at pH 7.4, the same pulsed NIR regimen facilitated a modest softening and degradation of the GelMA network (Figure 3I), which can be attributed to a temperature‐induced increase in hydrogel mesh mobility and solute transport, as reported for GelMA‐based systems [42]. Together, these results demonstrate that, under clinically relevant intermittent irradiation rather than continuous long‐term exposure, MXene@Cu‐MOF/GelMA enables controllable, mildly enhanced Cu^2+^ release and hydrogel relaxation, in line with the mild photothermal conditions employed in our in vivo studies.

Modeling of the 24 h cumulative release fraction as a function of pH revealed an S‐shaped dependence for the MXene@Cu‐MOF/GelMA platform (Figure 3J). For imidazolate/ZIF‐like nodes, literature shows acid‐sensitive dissolution in mildly acidic media (≈pH 6.0–5.0), whereas HKUST‐type (carboxylate) Cu‐MOFs are less stable in wet/acidic environments—together supporting the observed transition region and minimal release near neutrality [43]. Across pH, raising the temperature from 37 to 45°C shifted the curves upward, yielding a higher release fraction at a given pH, in line with Arrhenius‐type acceleration of acid‐catalyzed node cleavage under NIR [41].

The hydrogels exhibited a pH‐responsive release behavior, with markedly accelerated Cu^2+^ release at pH 6.8 compared with pH 7.4. After 24 h, the cumulative release reached approximately 1.85 µg/mL at pH 7.4, whereas ∼3.82 µg/mL was released at pH 6.8. At 72 h, the cumulative release approached ∼2.83 µg/mL and ∼4.93 µg/mL for pH 7.4 and pH 6.8, respectively, consistent with the partially acid‐labile coordination environment of the Cu‐MOF phase (Figure S10).

As shown in Figure S11, NIR‐induced photothermal effects further accelerate Cu^2+^ release, with a more pronounced enhancement at pH 6.8. For example, at 24 h, the Cu^2+^ concentration at pH 6.8 with NIR irradiation (+NIR) is approximately 4.78 µg/mL, which is significantly higher than that at pH 6.8 without NIR (‐NIR, ∼3.82 µg/mL). Similarly, at pH 7.4, the Cu^2+^ release under NIR (∼2.22 µg/mL) is also notably higher than that under dark conditions (∼1.85 µg/mL). These results are in agreement with our design concept of achieving on‐demand cascade activation under infection/inflammation‐related pathological microenvironments in combination with external NIR irradiation.

**FIGURE 4:**
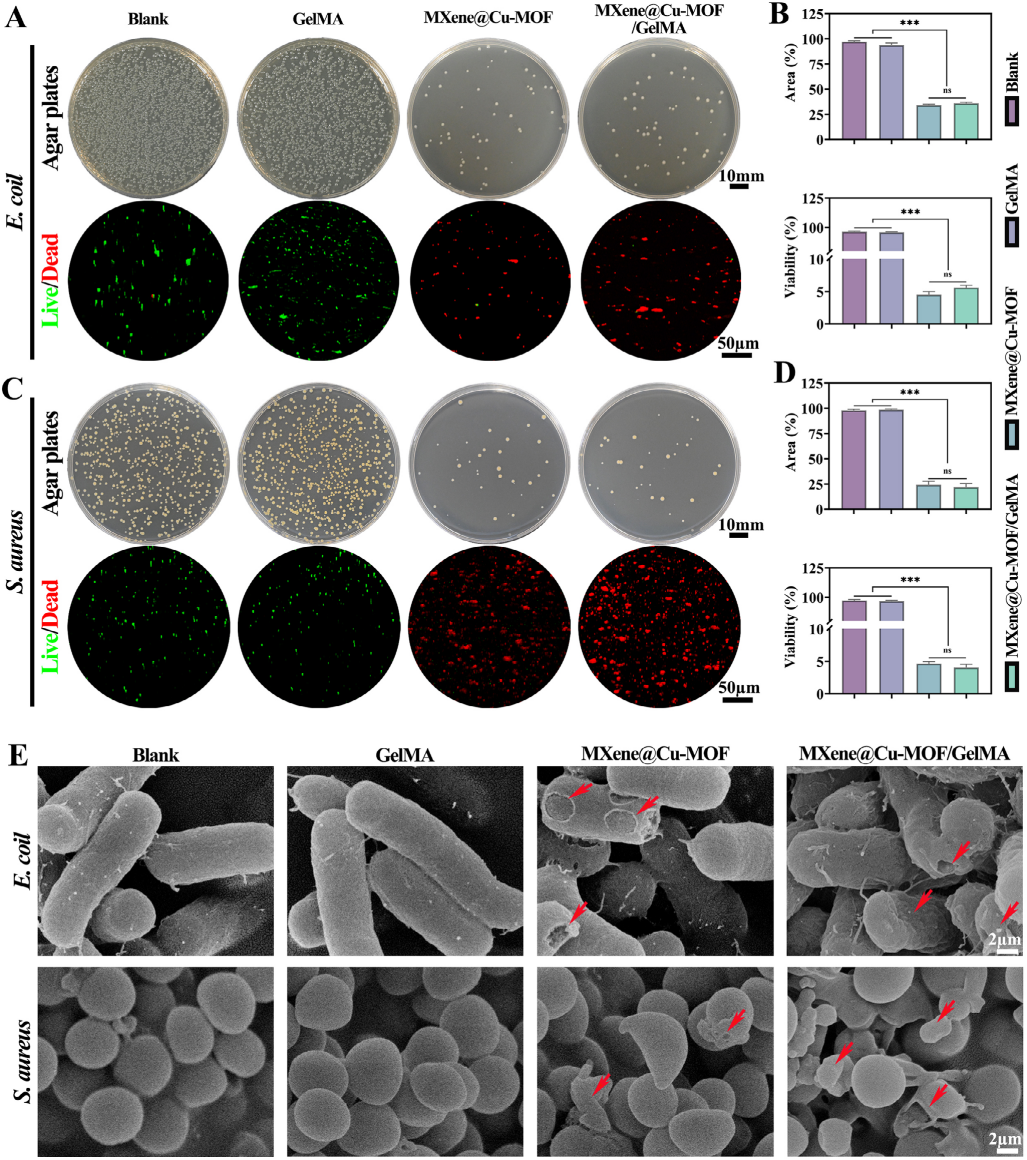
In vitro antibacterial activity of the MXene@Cu‐MOF/GelMA platform upon NIR against E. *coli* and S. *aureus*. A) Representative agar plate images of E. *coli* colonies and corresponding Live/Dead fluorescence staining after treatment with PBS (blank), GelMA, MXene@Cu‐MOF, and MXene@Cu‐MOF/GelMA. B) Quantification of E. *coli* colony area (plates) and percent viability (Live/Dead). C) Representative agar plate images of S. *aureus* colonies and corresponding Live/Dead staining under the same treatments as in (A). D) Quantification of S. *aureus* colony area and percent viability. E) Representative SEM micrographs of E. *coli* and S. *aureus* after the indicated treatments, with red arrows indicating damaged bacteria. Data are presented as mean ± SD (n = 3); ^***^ *p* < 0.001; ns, not significant.

**FIGURE 5:**
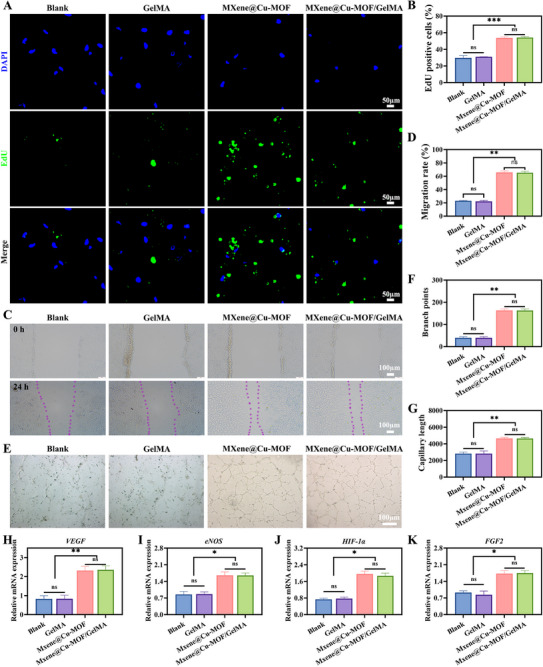
In vitro angiogenic assessment of the MXene@Cu‐MOF/GelMA platform upon NIR. A,B) EdU incorporation staining of HUVECs and quantification of the percentage of EdU^+^ nuclei in the following groups: Blank (PBS), GelMA, MXene@Cu‐MOF, and MXene@Cu‐MOF/GelMA. C,D) Scratch‐wound migration assay of HUVEC monolayers and quantification of wound closure (%) at the indicated time points. E–G) Matrigel tube‐formation assay: representative images E) with quantification of branch points (F) and total tube length (G). H–K) RT–qPCR analysis of angiogenesis‐related genes (*VEGF*, *eNOS*, *HIF‐1α*, and FGF2) in HUVECs. Data are presented as mean ± SD (n = 3); ^*^ *p* < 0.05, ^**^ *p* < 0.01, ^***^ *p* < 0.001; ns, not significant.

Acidification and temperature act in a cooperative manner. At neutral pH the system shows slow baseline leakage; mild acidification produces a marked increase in release (especially for imidazolate‐like nodes); further acidification or brief NIR exposure drives near‐burst output—an on‐demand behavior also reported for NIR‐responsive hydrogels and antibiofilm platforms [44]. These trends are consistent with the central design concept of this work: an integrated “skeleton–backpack” in which the MXene skeleton efficiently converts light to heat and the interfacial MOF “backpack” translates that heat into on‐demand ionic dosing. GelMA encapsulation preserves function by preventing nanosheet aggregation while allowing heat and small solutes to diffuse—consistent with GelMA's tunable viscoelasticity/transport properties [45]. Functionally, this photothermal switch constitutes the second stage of the cascade: after early oxidative‐stress control (Section 3.2), a short NIR dose delivers spatially and temporally co‐localized heat + Cu^2+^, underpinning enhanced antibiofilm activity (Figure 4) and leaving a later low‐dose Cu^2+^ background that supports angiogenesis and chondrogenesis (Figure 5); low‐level Cu^2+^ signaling in pro‐angiogenic contexts is also documented [46]. The interfacial chemistry of oxygen‐terminated MXene with metal/MOF nodes provides the structural basis for heat‐accelerated decomposition and controlled release in in situ‐grown MXene–MOF heterostructures [47].

### 3.5 Potent Antibacterial Activity upon NIR Against E. *coli* and S. *aureus*

On agar plates, both MXene@Cu‐MOF and MXene@Cu‐MOF/GelMA upon NIR (808 nm, 1.0 W/cm^2^, 10 min) yielded fewer and smaller colonies than PBS (Blank) and GelMA for E. *coli* and S. *aureus* (Figure 4A–D; mean ± SD, n = 3). Live/Dead staining showed fields dominated by nonviable (red) bacteria in the two active groups, whereas Blank and GelMA were dominated by viable (green) cells. Quantitative analysis from plate counts and fluorescence images showed a significant reduction in colony area and bacterial viability for MXene@Cu‐MOF and MXene@Cu‐MOF/GelMA compared with controls, while no significant difference was detected between these two active groups under the NIR conditions tested. SEM corroborated these outcomes, revealing severe envelope damage. In E. *coli*, we observed surface pitting, corrugation, and cell lysis; in S. *aureus*, we noted wall rupture, shrinkage or collapse, and membrane debris (Figure 4E). Such photothermal antibacterial behavior of MXene‐containing hydrogels and composites under NIR has been widely reported [48].

It should be noted that a small number of residual colonies could still be detected on the agar plates after NIR treatment, because the antibacterial assay involves 24 h incubation in a nutrient‐rich medium and can thus amplify even a very small fraction of surviving bacteria into visible colonies. In contrast, the Live/Dead staining reflects the instantaneous viability of bacteria on the material surface immediately after treatment and does not capture subsequent regrowth in fresh medium. Thus, the high sensitivity of plate counting for residual survivors and the more intuitive “instant killing” impression from fluorescence images should be viewed as complementary and not contradictory readouts of the same antibacterial response.

These results are consistent with the platform's designed antibacterial mechanism under NIR. The pH‐responsive MOF “backpack” releases Cu^2+^ that mediate chemical killing, while the 2D MXene scaffold presents a broad contact interface that helps confine bacteria near the composite surface and increases local exposure to Cu^2+^. Cu‐based antimicrobials are known to be broad‐spectrum via membrane disruption and redox chemistry [49]. Encapsulation in GelMA does not blunt efficacy, in agreement with Sections 3.2 and 3.4, because the hydrogel mesh permits rapid diffusion of small ions yet prevents secondary aggregation of the heterostructure [50]. This explains why the dispersion and the hydrogel composite perform comparably in planktonic and colonial assays.

Functionally, this baseline antibacterial capacity forms the second stage of the cascade after early oxidative‐stress control. NIR light was applied in the experiments the heterostructure is primed for on‐demand amplification: photothermal heating by the MXene skeleton accelerates MOF breakdown and drives a burst of Cu^2+^ release at the bacteria–material interface, producing co‐localized heat and ions that is particularly relevant for biofilm contexts (Section 3.4 and Figure 4). This mechanism aligns with reports that Cu^2+^ provides broad‐spectrum antimicrobial activity and that photothermal inputs can potentiate antibiofilm efficacy when appropriately dosed [51]. In contrast to physical mixtures, the unified heterostructure couples heat generation and ionic delivery through a single interface, offering a practical route from routine antibacterial control to more aggressive antibiofilm therapy when infection pressure escalates [52].

### 3.6 Biocompatibility of Individual Components and Composite Hydrogels

Chondrocytes play a central role in tracheal cartilage regeneration, as the native tracheal wall relies on hyaline cartilage rings populated by chondrocytes to maintain airway rigidity and prevent collapse. Accordingly, tissue‐engineered tracheal grafts must provide a microenvironment that supports chondrocyte survival, spreading, and matrix production over time. Biocompatibility of the scaffold is therefore a key design criterion in tracheal tissue engineering, ensuring that the material does not induce cytotoxicity while enabling robust cell adhesion and proliferation. GelMA‐based hydrogels are widely recognized as biocompatible matrices for chondrocytes and cartilage repair, owing to their cell‐adhesive motifs and cartilage‐like ECM compatibility.

In this context, we systematically assessed the cytocompatibility of GelMA, MXene@Cu‐MOF, and MXene@Cu‐MOF/GelMA using primary chondrocytes. Live/Dead staining at days 1, 4, and 7 showed predominantly viable (green) cells in all groups (Blank, GelMA, MXene@Cu‐MOF, and MXene@Cu‐MOF/GelMA), with no obvious increase in dead (red) cells in the MXene‐ or MXene@Cu‐MOF‐containing samples compared with controls (Figure S12A). Consistently, F‐actin/DAPI staining revealed well‐spread cytoskeletons and clear actin stress fibers across all groups, indicating that chondrocytes could adhere and extend on the surfaces of the composite hydrogels without evident morphological signs of stress or apoptosis (Figure S12B).

CCK‐8 quantification further showed that cell viability in all groups remained above 95%, with no obvious differences among the groups (Figure S12C). In addition, the proliferative activity (OD values) increased markedly from day 1 to day 7 in all groups, and no significant differences in proliferation were observed between them (Figure S12D), indicating that the incorporation of MXene and Cu‐MOF did not compromise the intrinsic cytocompatibility of GelMA.

Taken together, these results suggest that the MXene@Cu‐MOF/GelMA hydrogel effectively supports chondrocyte adhesion, spreading, and proliferation, with a cytocompatibility comparable to that of pure GelMA, and thus represents a promising scaffold candidate for tracheal cartilage repair.

### 3.7 Pro‐Angiogenic Responses of Endothelial Cells In Vitro under NIR Irradiation

All experimental groups in this section were subjected to NIR irradiation, consistent with reports that MXene‐based photothermal hydrogels enable mild‐hyperthermia–assisted tissue repair and vascularization [53]. In HUVEC assays, MXene@Cu‐MOF/GelMA consistently showed the strongest pro‐angiogenic phenotype across all readouts. Representative micrographs showed a higher density of EdU‐positive nuclei in the MXene@Cu‐MOF and MXene@Cu‐MOF/GelMA groups than in Blank (PBS), or GelMA. Quantitative analysis confirmed a stepwise pattern, with the MXene@Cu‐MOF and MXene@Cu‐MOF/GelMA outperforming GelMA and Blank (Figure 5A,B). In scratch‐wound assays, the MXene@Cu‐MOF and MXene@Cu‐MOF/GelMA accelerated wound closure at matched time points, and GelMA and Blank closed more slowly (Figure 5C,D), consistent with literature showing that mild hyperthermia enhances endothelial motility [54]. On Matrigel, the MXene@Cu‐MOF and MXene@Cu‐MOF/GelMA generated denser and more interconnected capillary‐like networks, with more branch points and a greater total tube length than all other groups (Figure 5E–G). Consistent with these functional gains, RT‐qPCR revealed up‐regulation of *VEGF*, *eNOS*, *HIF‐1α*, and *FGF2* in the MXene@Cu‐MOF and MXene@Cu‐MOF/GelMA groups relative to controls (Figure 5H–K), in line with evidence that Cu^2+^ activate HIF‐1α and downstream VEGF/eNOS signaling to promote angiogenesis [55]. Across assays, cell morphology and coverage did not suggest overt cytotoxicity under the tested conditions, aligning with reports of the biocompatibility of MXene‐containing hydrogels and dressings [53].

These findings align with the cascade‐programmed design and indicate that, after early control of oxidative stress and infection, the platform transitions into a regeneration‐supportive state. Beyond NIR excitation, the platform can also furnish a low and sustained level of Cu^2+^, a regime reported to stabilize hypoxia‐related signaling and to enhance VEGF and eNOS pathways, thereby promoting endothelial proliferation, migration, and tubulogenesis [55]. The concordant up‐regulation of *VEGF*, *eNOS*, *HIF‐1α*, and *FGF2* matches this pathway logic and mirrors the in vivo transcriptomic enrichment of angiogenic programs (Figure 8). The GelMA matrix provides cell‐adhesive motifs and a hydrated mesh that support endothelial attachment and sprouting, as summarized in recent vascularization‐focused hydrogel reviews and studies [56]. Meanwhile, the MXene backbone offers a nano‐textured interface and helps maintain a low‐oxidative‐stress microenvironment (Section 3.2), consistent with MXene's documented ROS‐scavenging and redox‐modulating properties—removing a known brake on angiogenesis [57]. This view is also aligned with emerging immuno‐supportive hydrogel strategies showing that modulating oxidative stress and endothelial crosstalk under NIR‐induced mild hyperthermia can enhance repair [58]. That MXene@Cu‐MOF/GelMA outperforms either component alone further indicates true synergy rather than additive behavior: MXene preserves redox balance and presentation, Cu‐MOF provides the time‐programmed pro‐angiogenic cue, and GelMA stabilizes dispersion without passivating function—an integration echoed by recent MXene‐enabled photothermal frameworks for infection control and regeneration [59].

### 3.8 In Vitro Cartilage Formation and Subcutaneous Maturation of TET

**FIGURE 6:**
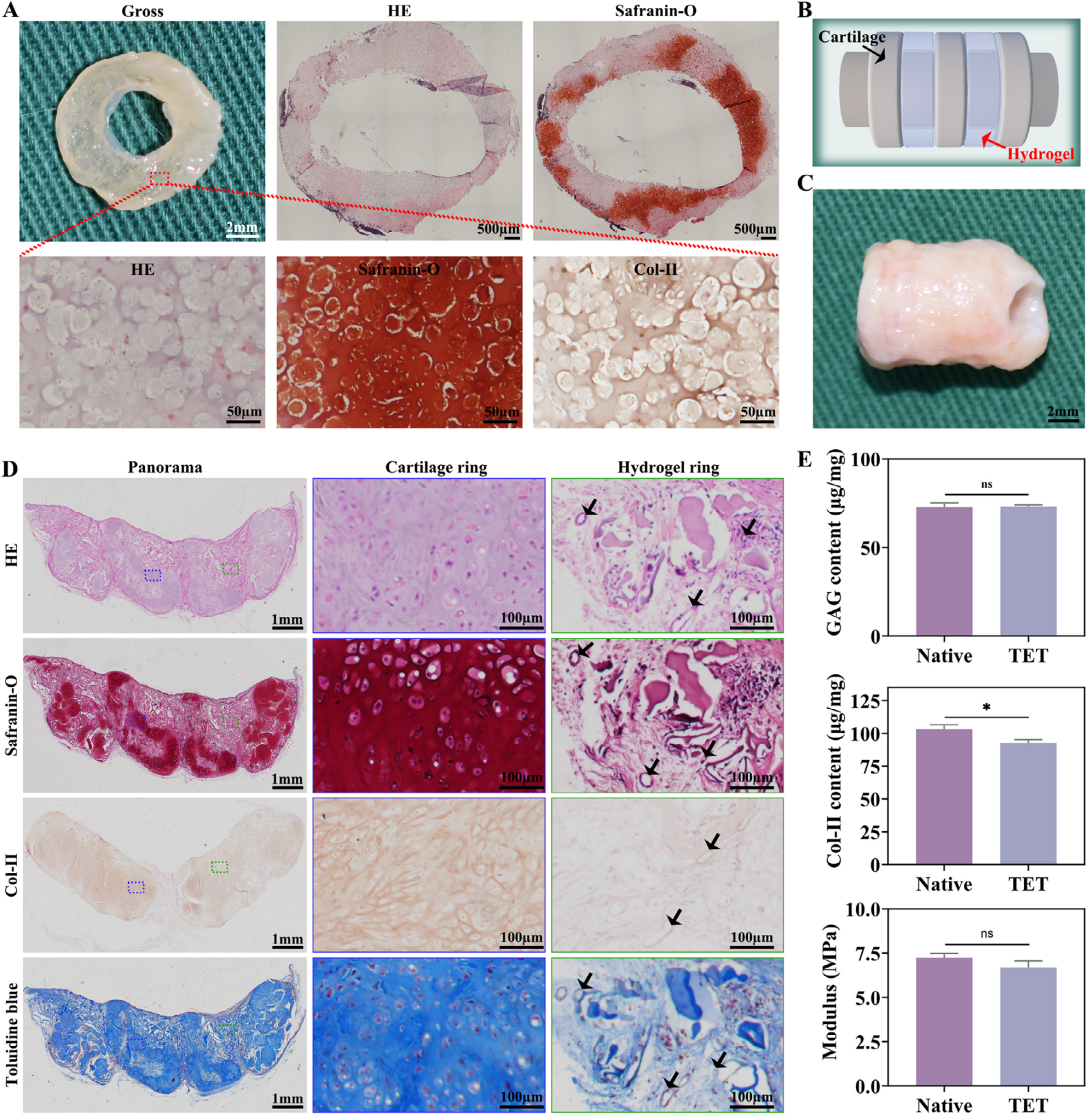
Reconstruction of TET in vitro and after 4‐week subcutaneous implantation in nude mice. A) Gross photographs and histology of in vitro–cultured cartilaginous rings at 4 weeks: HE, Safranin‐O, and immunohistochemistry for Col‐II. Lower panels show magnified views of the boxed regions. B) Schematic illustration of TET fabrication. C) Gross photograph of regenerated TET after 4 weeks of subcutaneous implantation in nude mice. D) Histological evaluation of explanted TET: HE, Safranin‐O, immunohistochemical Col‐II, and Toluidine Blue staining; black arrows indicate blood vessels. E) Quantitative analyses of GAG content, Col‐II content, and compressive Young's modulus of regenerated TET vs. native rabbit trachea. Data are presented as mean ± SD (n = 3); ^*^ *p* < 0.05; ns, not significant.

To further evaluate cartilage formation both in vitro and in vivo, gross morphology, HE staining, Safranin‐O staining, and immunohistochemical Col‐II staining were performed. As shown in Figure 6A, the regenerated cartilage in vitro appeared milky white, smooth, and slightly translucent, with a dense texture. Histological images revealed that HE staining displayed plump chondrocytes with clearly defined nuclei and typical lacunae structures. Safranin‐O staining demonstrated abundant distribution of GAG. Col‐II staining was strongly positive, indicating that the chondrocytes within the MXene@Cu‐MOF/GelMA retained a stable phenotype and that the regenerated tissue possessed cartilage‐like characteristics.

Figure 6B shows a schematic diagram of the in vitro assembled TET model. The gross appearance of the subcutaneously implanted TET after 4 weeks in nude mice (Figure 6C) revealed a well‐defined tubular structure, with alternating cartilaginous and connective tissue rings [1]. The MXene@Cu‐MOF/GelMA surface was uniformly covered with neotissue, and the regenerated cartilage exhibited an ivory‐white color with cartilage‐like texture and elasticity. HE staining demonstrated evenly distributed chondrocyte‐like cells with rounded morphology and compact arrangement. In some regions, typical lacunar structures were observed, indicating a relatively high degree of cartilage maturation. Col‐II staining was strongly positive, suggesting the presence of cartilage‐specific ECM. Safranin‐O and Toluidine blue staining further confirmed abundant and uniformly distributed GAG, supporting cartilage phenotype (Figure 6D). Quantitative analysis showed negligible differences in GAG and Col‐II content between the regenerated TET and native tracheal cartilage. The Young's modulus of the regenerated TET was slightly less than that of native cartilage, indicating similar mechanical properties (Figure 6E).

The expression of key cartilage‐specific markers was examined at both the protein and gene levels. Western blotting showed that the MXene@Cu‐MOF and MXene@Cu‐MOF/GelMA groups exhibited stronger bands for Aggrecan and Col‐II compared with the Blank and GelMA groups (Figure S13A). Semi‐quantitative analysis revealed that the relative protein levels of Aggrecan and Col‐II were significantly higher in the MXene@Cu‐MOF and MXene@Cu‐MOF/GelMA groups than in the Blank/GelMA groups, whereas no significant difference was observed between MXene@Cu‐MOF and MXene@Cu‐MOF/GelMA (Figure S13B). Consistently, qRT‐PCR analysis demonstrated that the mRNA expression of ACAN and COL2A1 followed a similar trend (Figure S13C). Both MXene@Cu‐MOF and MXene@Cu‐MOF/GelMA markedly upregulated ACAN and COL2A1 expression relative to Blank and GelMA, while the difference between MXene@Cu‐MOF and MXene@Cu‐MOF/GelMA was not statistically significant. These results indicate that the chondrogenic performance of MXene@Cu‐MOF/GelMA is significantly superior to that of MXene/GelMA.

These outcomes indicate that the MXene@Cu‐MOF/GelMA establishes a pro‐chondrogenic niche while still permitting vascular ingrowth during subcutaneous maturation—an otherwise difficult balance in airway constructs [11]. Mechanistically, early control of oxidative stress and bacterial burden (Sections 3.2–3.5) likely preserves chondrocyte viability and supports matrix synthesis, enabling mechanical properties to approach physiological levels without compromising tissue integration; this agrees with reports that mitigating inflammatory/oxidative cues improves cartilage formation and graft stability [60]. In our system, the unified heterostructure helps sustain this balance: MXene maintains a low‐oxidative microenvironment via ROS scavenging [61], the MOF “backpack” provides time‐programmed Cu^2+^ that supports revascularization without excessive dosing [62], and the GelMA matrix stabilizes dispersion and presents a hydrated, cell‐adhesive network favorable for chondrogenesis [63]. Together, these features link microenvironment control to improved cartilage matrix deposition and mechanical function, positioning the platform for the subsequent orthotopic tracheal repair studies.

### 3.9 Orthotopic Repair of Extensive Tracheal Defects in Rabbits

**FIGURE 7:**
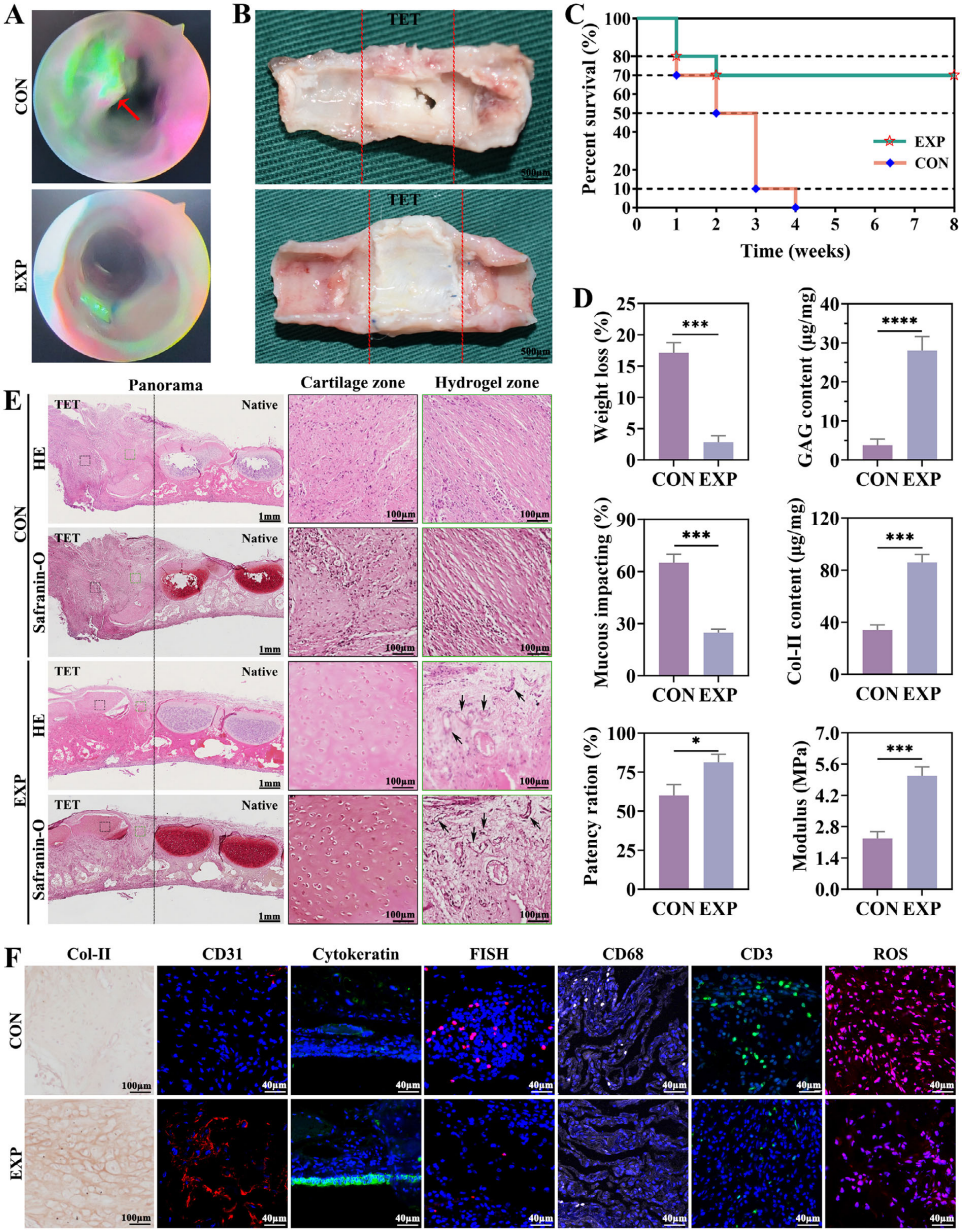
Therapeutic efficacy of TET in an orthotopic extensive tracheal defect rabbit model. A,B) Bronchoscopic views and gross morphology of repaired tracheae in control (CON) and experimental (EXP) groups at 4 weeks; Red arrow indicates hyperplasia and dotted red lines outline repaired TET. C) Kaplan–Meier survival curves over 8 weeks post‐surgery. D) Quantitative outcomes at 2 weeks: body weight change, tracheal patency ratio, mucous‐impaction score, GAG content, Col‐II content, and compressive Young's modulus. E) Histology of regenerated tracheal tissues at 4 weeks: HE and Safranin‐O staining, with higher‐magnification insets highlighting cartilage regeneration and hydrogel integration; blank arrows indicate vessels and dotted black lines outline repaired TET and native tracheal tissue, black squares outline cartilage zone and green squares outline hydrogel zone. F) Tissue staining at 4 weeks: immunohistochemistry for Col‐II, CD31 (angiogenesis marker), cytokeratin (epithelialization marker), CD68 (macrophages), and CD3 (T cells), together with FISH for bacterial burden and ROS staining for oxidative stress. Data are presented as mean ± SD (n = 3); ^*^ *p* < 0.05, ^***^ *p* < 0.001, ^****^ *p* < 0.0001; ns, not significant.

In the orthotopic rabbit model, bronchoscopy showed wider and better‐perfused lumens with fewer adherent mucus plaques in the experimental group (EXP; MXene@Cu‐MOF/GelMA engineered TET) than in controls (CON; single GelMA engineered TET) at all observed time points (Figure 7A). Gross examination confirmed a more circular, unobstructed airway in EXP, whereas CON frequently displayed segmental narrowing and destruction (Figure 7B). Kaplan–Meier analysis indicated higher survival in EXP over eight weeks (Figure 7C).

At two weeks, quantitative readouts favored EXP. Compared with CON, EXP showed smaller postoperative weight loss, a higher tracheal patency ratio, lower mucus‐impaction scores, and improved tissue‐quality metrics, including higher GAG and Col‐II contents and a greater compressive Young's modulus (Figure 7D; mean ± SD, n = 3). By four weeks, histology demonstrated more continuous cartilage formation spanning the graft with clear scaffold integration (Figure 7E). Multiplex staining at four weeks further showed, in EXP, higher Col‐II deposition and increased CD31‐positive microvessel density (angiogenesis), more continuous cytokeratin‐positive epithelial coverage (epithelialization), a lower bacterial burden by fluorescence in situ hybridization, and attenuated inflammation with fewer CD68‐positive macrophages and CD3‐positive T cells, together with a reduced tissue reactive‐oxygen signal, relative to CON (Figure 7F).

To further evaluate the pro‐angiogenic capacity of the different scaffolds in vivo, CD31 immunohistochemistry was performed on tissue sections surrounding the implants. As shown in Figure S14A, only a few CD31^+^ microvessels were observed in the Blank and GelMA groups, whereas MXene@Cu‐MOF led to a moderate increase in microvessel formation. In striking contrast, the MXene@Cu‐MOF/GelMA group exhibited markedly higher densities of CD31^+^ vessels with more mature and interconnected microvascular networks. Quantitative analysis of CD31^+^ vessel number per field (Figure S14B) confirmed that MXene@Cu‐MOF/GelMA significantly enhanced neovascularization compared with Blank, GelMA, and MXene@Cu‐MOF, whereas no significant difference was detected between the Blank and GelMA groups. These results demonstrate that the cascade‐responsive MXene@Cu‐MOF/GelMA hydrogel provides a more favorable microenvironment for angiogenesis than MXene/GelMA and GelMA alone.

At week 4 after implantation, no significant abnormalities were observed in the serum biochemical indices of the hydrogel‐treated group compared with the control group (Figure S15A), and histological sections of the major organs exhibited no obvious tissue damage or abnormal inflammatory infiltration (Figure S15B). These findings indicate that the long‐term retention of copper in vivo did not result in detectable systemic toxicity.

To evaluate the biodistribution and clearance of copper, Cu^2+^ levels in serum and major organs were quantified by ICP‐MS. As shown in Figure S16A, serum Cu^2+^ concentrations in the experimental group (EXP) at 1 and 4 weeks post‐surgery were comparable to those in the control group (CON), remaining at approximately 8–10 µg/mL with no statistically significant differences between groups. Similarly, the Cu^2+^ content in major organs, including the heart, liver, spleen, lung, and kidney (Figure S16B), was essentially identical between CON and EXP groups. The liver exhibited the highest Cu^2+^ level, consistent with its physiological role in trace metal storage, whereas the other organs showed only low background signals without obvious elevation in the EXP group. In addition, the Cu^2+^ levels measured in these organs were close to previously reported values for healthy rabbits and fell within the normal range [64]. Collectively, these data indicate that the released Cu^2+^ does not undergo long‐term accumulation in distant organs, and this biodistribution profile supports the good systemic safety of the MXene@Cu‐MOF/GelMA hydrogel within the observation period.

These outcomes translate the platform's cascade logic into functional tracheal repair. Early control of oxidative stress (Section 3.2) likely blunted the postoperative inflammatory surge, limiting excessive granulation and fibrotic stenosis, which is consistent with the lower reactive‐oxygen signal and reduced macrophage and T‐cell infiltration [65]. The composite's baseline antibacterial capacity—through pH‐responsive copper release without exogenous triggers—aligns with the lower bacterial signal and reduced mucus impaction, interrupting the cycle of infection, inflammation, and mucus stasis [55]. As inflammation subsided, the platform entered a regeneration‐supportive phase: sustained low‐dose copper supported microvascular ingrowth and epithelial continuity, providing perfusion and barrier functions that stabilize the lumen [66]. The increases in GAG and Col‐II and the improved stiffness indicate cartilage‐like matrix maturation, which underpins long‐term patency.

Importantly, these benefits were achieved with the MXene@Cu‐MOF/GelMA engineered TET under baseline conditions, while the material retains an on‐demand NIR option (Section 3.4) for situations of escalating infection risk, offering a clinically flexible control point [67]. Compared with prior tracheal graft strategies that often trade off infection control against revascularization and tissue maturation, the unified heterostructure couples photothermal conversion and controlled ionic dosing within a single, shape‐conformable hydrogel scaffold [68]. This integration enables simultaneous modulation of infection and inflammation together with vascular–epithelial–cartilage reconstruction, addressing core hurdles in extensive tracheal defect repair and aligning with recent and classical observations in airway tissue engineering.

### 3.10 Systems‐Level Transcriptomic Corroboration

**FIGURE 8:**
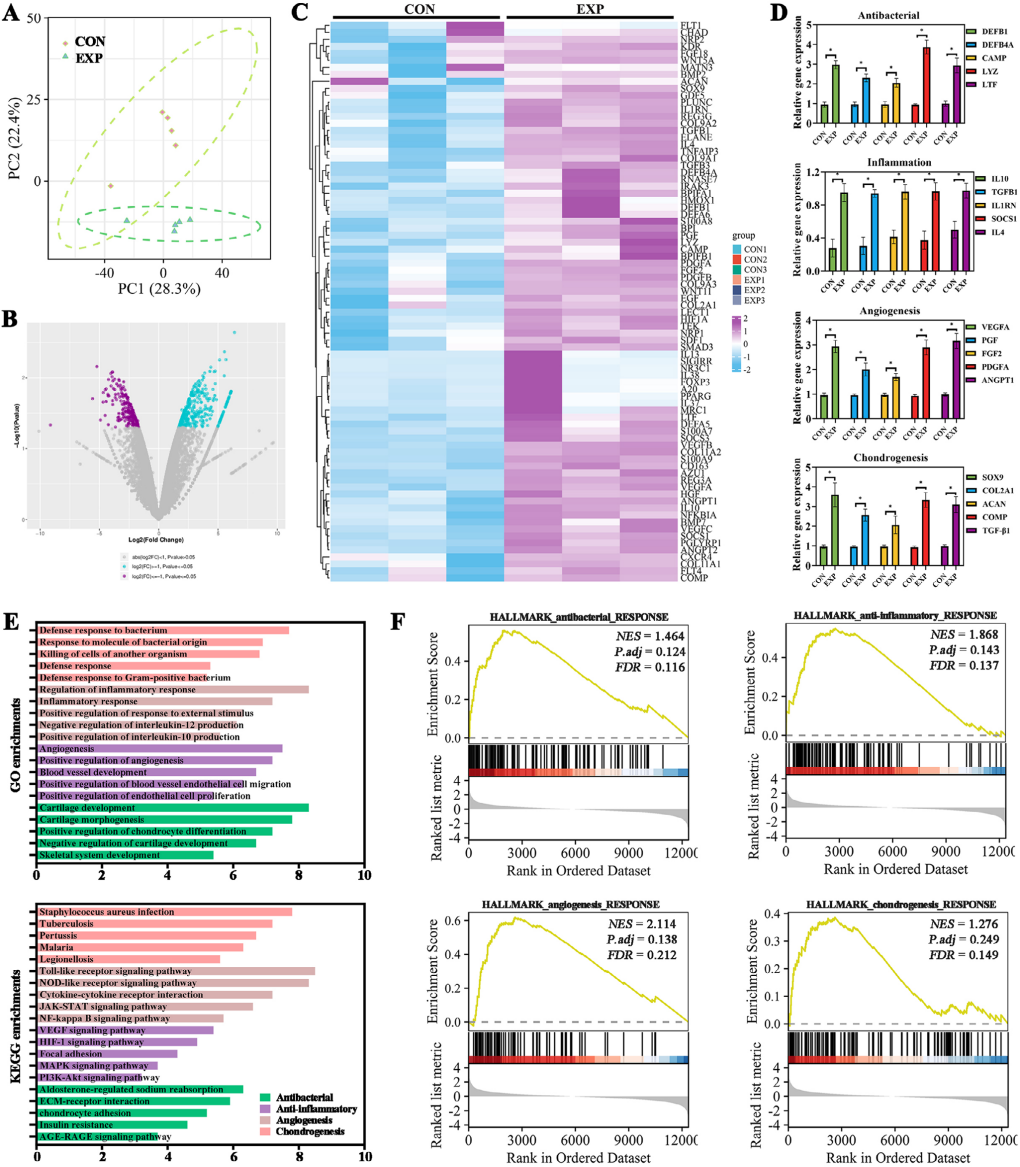
Whole‐transcriptome RNA‐seq analysis of repaired tracheal grafts at 2 weeks. A) PCA of global transcriptomic profiles for CON and EXP groups. B) Volcano plot of DEGs between EXP and CON; significance defined as |log_2_ fold change| ≥ 1 (fold change ≥ 2) and Benjamini–Hochberg–adjusted *p* < 0.05. C) Heatmap of significant DEGs across samples, illustrating relative expression patterns between groups. D) Relative expression of representative genes associated with antibacterial defense, inflammatory regulation, angiogenesis, and chondrogenesis in CON vs. EXP. E) GO and KEGG pathway enrichment analyses of genes upregulated in EXP vs. CON (top enriched terms are shown). F) GSEA enrichment plots for selected gene sets related to antibacterial/immune defense, inflammatory response, angiogenesis, and chondrogenic programs, showing positive enrichment in EXP relative to CON. Data are presented as mean ± SD (n = 3); ^*^ *p* < 0.05.

Bulk RNA‐seq of repaired tracheal tissues at 2 weeks revealed clear group separation. PCA clustered all EXP samples apart from CON along the major axes, indicating a global transcriptional shift associated with the composite platform (Figure 8A). Volcano plots identified a substantial set of DEGs that met conventional cutoffs for significance (adjusted *p* < 0.05 and at least twofold change), and sample‐wise heatmaps showed tight intra‐group coherence with consistent up‐ and down‐regulation patterns across biological replicates (Figure 8B,C).

Functional summaries converged on four pro‐repair program blocks elevated in EXP: antibacterial defense, anti‐inflammatory regulation, angiogenesis, and chondrogenesis (Figure 8D). GO and KEGG enrichment analyses highlighted pathways related to containment of innate stimuli and bacterial killing (including complement and lysosomal functions), endothelial activation and vessel development (angiogenesis and nitric‐oxide signaling), ECM organization (collagen fibrillogenesis and proteoglycan metabolism), and cartilage development (matrix assembly and chondrogenic differentiation) (Figure 8E). GSEA further demonstrated positive enrichment of curated signatures linked to antibacterial defense, resolution of inflammation, angiogenesis, and chondrogenic programs in EXP versus CON (Figure 8F).

While bulk RNA‐seq averages across cell types and cannot resolve cellular provenance, the strong replicate coherence and agreement with phenotype‐level endpoints argue against sampling artifacts. Future single‐cell or spatial transcriptomics could delineate contributions from endothelial, chondrocytic, epithelial, and immune compartments and refine the map of cell‐type–specific responders [69]. Overall, the RNA‐seq data provide systems‐level validation of the three‐stage cascade and support the mechanism‐linked efficacy of the MXene@Cu‐MOF platform in extensive tracheal repair.

### 3.11 Overall Interpretation and Outlook

Across models and scales, MXene@Cu‐MOF/GelMA functions as a single, structure‐integrated nanoplatform that (i) quenches ROS to prevent inflammatory escalation, (ii) delivers on‐demand, thermally amplified Cu^2+^ bursts to eradicate bacteria and biofilms, and (iii) maintains low‐dose Cu^2+^ to stimulate angiogenesis and support chondrogenesis. The true synergy arises from causal coupling—photothermal heating directly accelerates MOF degradation and ion release—rather than from additive effects of co‐loaded agents.

Limitations include the need to (i) define in‐vivo Cu exposure windows and long‐term metal clearance, (ii) map thermal dose in thick tissues, and (iii) optimize hydrogel degradation vis‐à‐vis airway biomechanics. Future studies can incorporate feedback control of NIR dosing, explore copper‐sparing MOF chemistries, and expand to infected large‐animal airway models.

## 4 Conclusion

This study introduces a self‐degradable messenger–enabled, cascade‐responsive MXene@Cu‐MOF heterostructure that converts a physical blend into a unified 2D/3D “skeleton–backpack” construct with causal coupling between MXene photothermal heating and MOF‐mediated Cu^2+^ release. The innovation lies in (i) the self‐degradable messenger paradigm—Cu‐MOF on MXene that basally releases Cu^2+^ under acidic/inflammatory cues and, upon NIR, undergoes burst, on‐demand ion release—and (ii) the cascade logic encoded in a single material: early ROS quenching, followed by photothermal effect against bacteria, then a low‐dose Cu^2+^ phase that drives angiogenesis and supports chondrogenesis. By in situ growth of MOF on Ti_3_C_2_T_x_, the heterostructure prevents restacking/aggregation, maximizes interfacial area, and ensures spatiotemporally co‐localized heat–ion delivery. Multiscale characterization, in vitro antioxidant/antibacterial/angiogenic assays, orthotopic rabbit tracheal repair, and transcriptomic analyses consistently validate both mechanism and efficacy. This work establishes a generalizable blueprint for on‐demand biointerfaces in oxidative injury, infection‐prone, poorly vascularized airway defects.

## Conflicts of Interest

The authors declare no conflict of interest.

## Supporting information




**Supporting File**: advs74059‐sup‐0001‐SuppMat.pdf

## Data Availability

The data that support the findings of this study are available from the corresponding author upon reasonable request.
