# Supplementary material for: Cascade‐Responsive MXene@Cu‐MOF Heterostructure Integrates Antioxidant Activity, Infection Control, and Vascularization for Tracheal Repair
Source: Adv Sci (Weinh). 2026 Jan 27;13(28):e21174. doi: 10.1002/advs.202521174 (PMC13185874; doi:10.1002/advs.202521174)
Supplement: Supplementary file 1 — Supporting File: advs74059‐sup‐0001‐SuppMat.pdf [file ADVS-13-e21174-s001.docx]

**Supporting Information**

**Cascade-Responsive MXene@Cu-MOF Heterostructure Integrates** **Antioxidant Activity, Infection Control, and Vascularization for Tracheal Repair**

*Liang Guo^1a^, Yingran Shen^1a^, Jiaoyu Yi^2a^, Juanjuan Li^3a^, Ziming Wang^1^, Erji Gao^1^, Siqiang Zheng^1^, Zhe-Sheng Chen^4^, Bo Tao^1*^*

^1^ Department of Thoracic Surgery, Shanghai Pulmonary Hospital, Tongji University, School of Medicine, Shanghai 200443, China

^2^ Department of Plastic Surgery, Renji Hospital, Shanghai Jiaotong University, School of Medicine, Shanghai 200127, China

^3^ Department of Medical Oncology, Shanghai Pulmonary Hospital and Thoracic Cancer Institute, Tongji University, School of Medicine, Shanghai 200443, China

^4^ Department of Pharmaceutical Science, College of Pharmacy and Health Science, St John’s University, Queens, New York 11439, United States of America

^a^ These authors contributed equally to this work.

*Corresponding to: Dr. Bo Tao, Email: tbo0820@tongji.edu.cn.

**
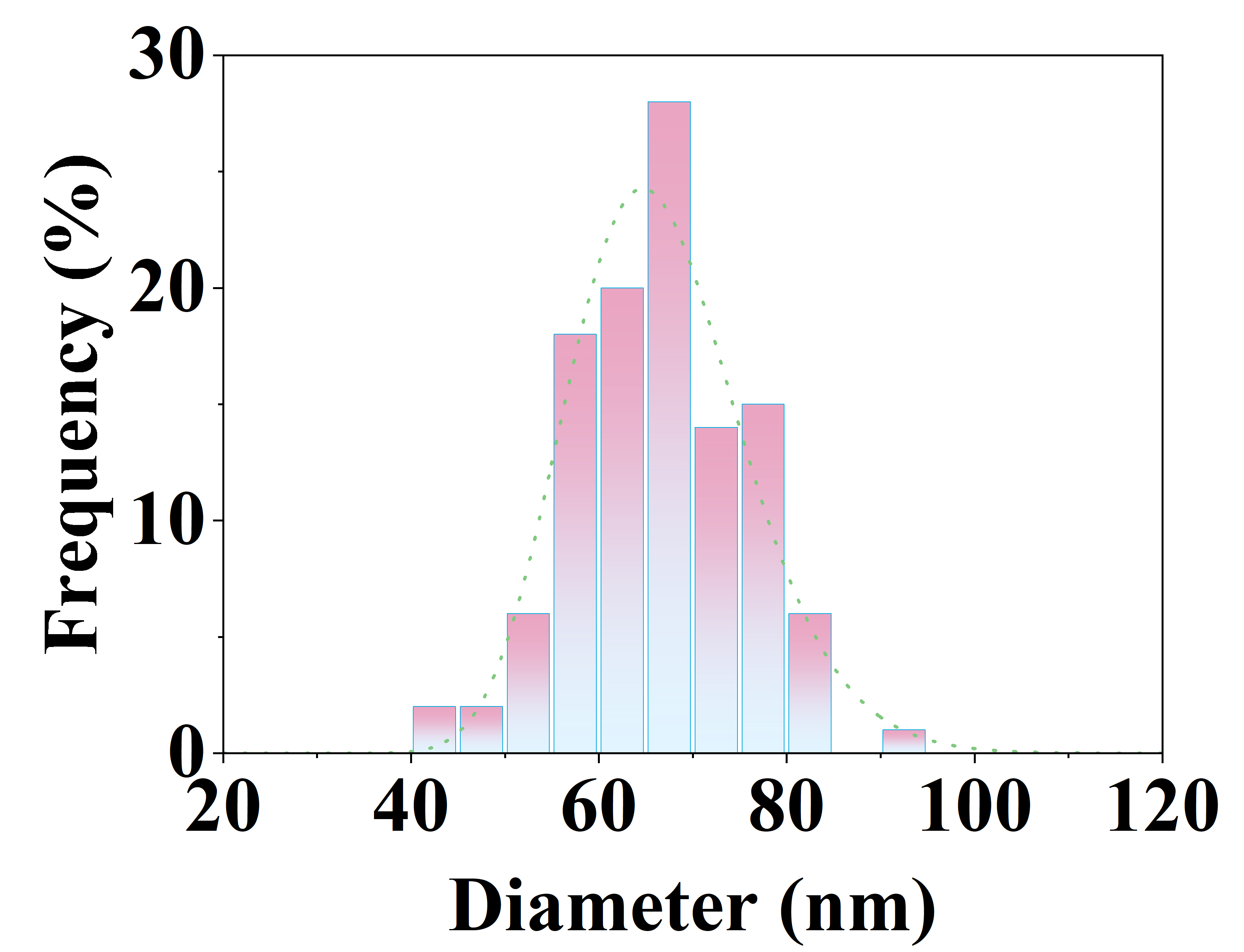
**

**Figure S1. Size distribution of Cu-MOF nanocrystals.** DLS intensity-weighted hydrodynamic diameter distribution of Cu-MOF dispersed in aqueous medium.

**
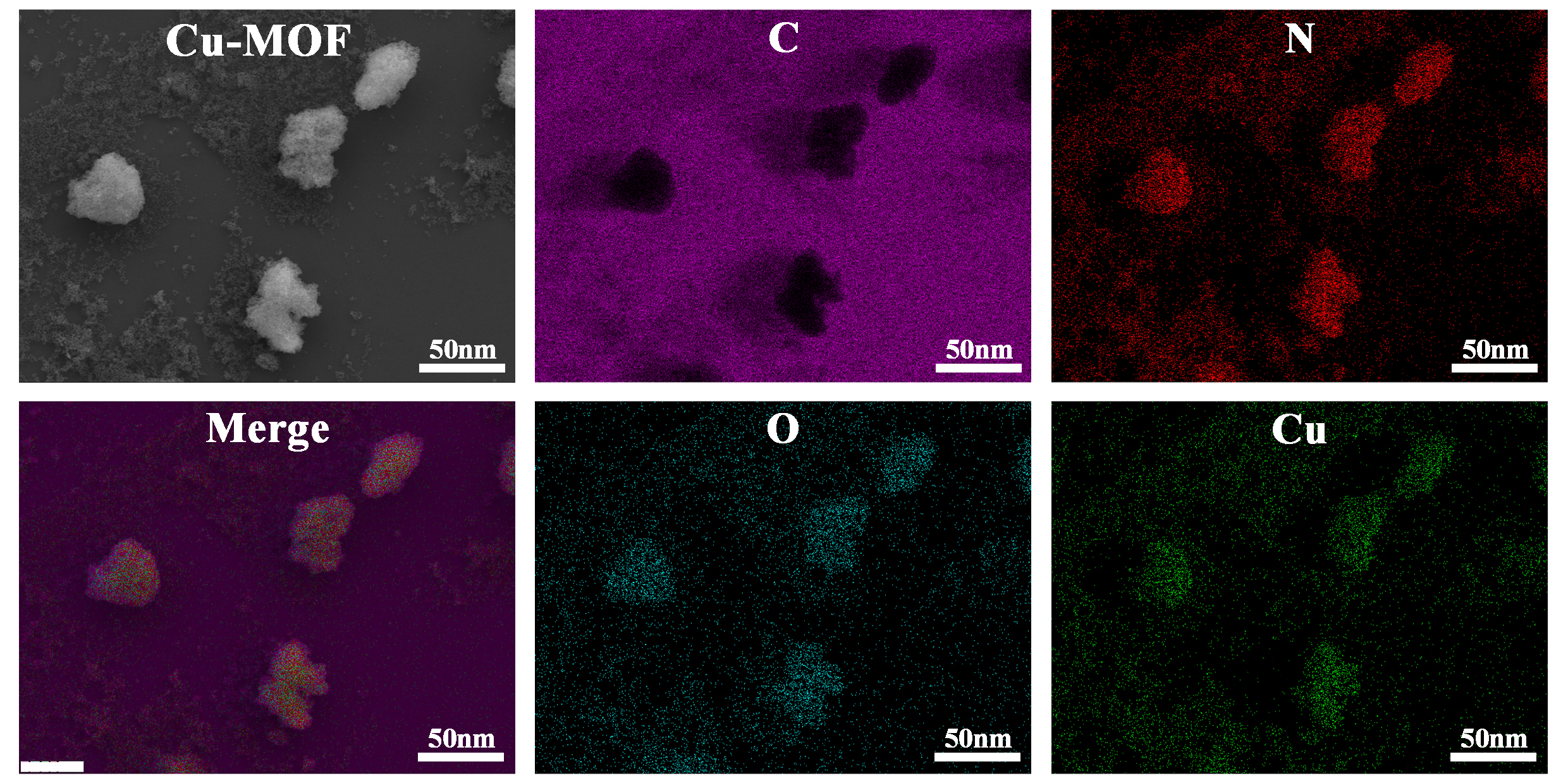
**

**Figure S2. EDS elemental mapping of Cu-MOF.** Representative SEM image and corresponding EDS elemental maps showing the spatial distribution of C, N, O, and Cu in Cu-MOF.

**
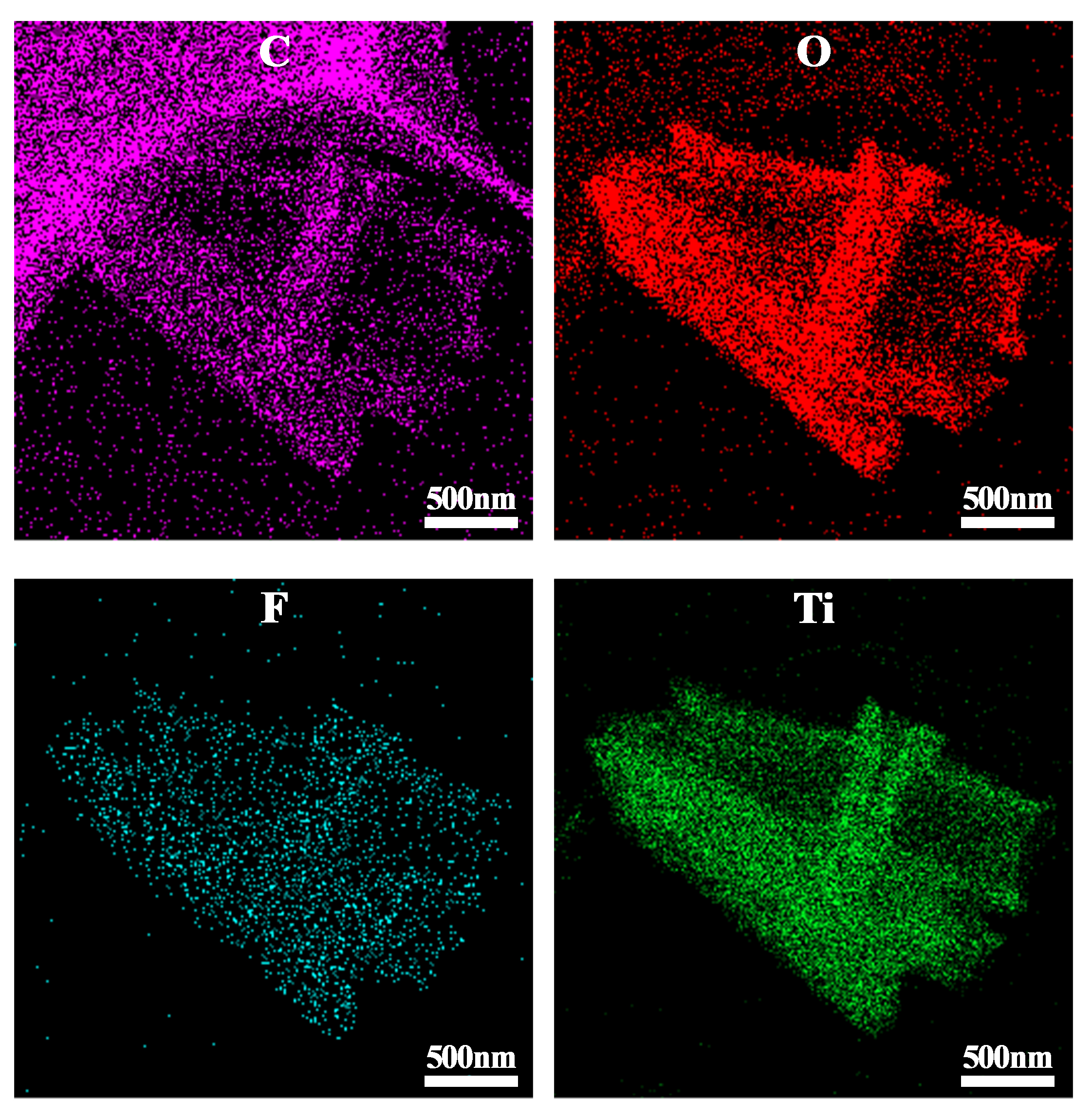
**

**Figure S3. EDS elemental mapping of MXene nanosheets.** Representative EDS elemental maps of MXene (Ti_3_C_2_T_x_), showing the spatial distributions of Ti, C, O, and F (surface terminations).


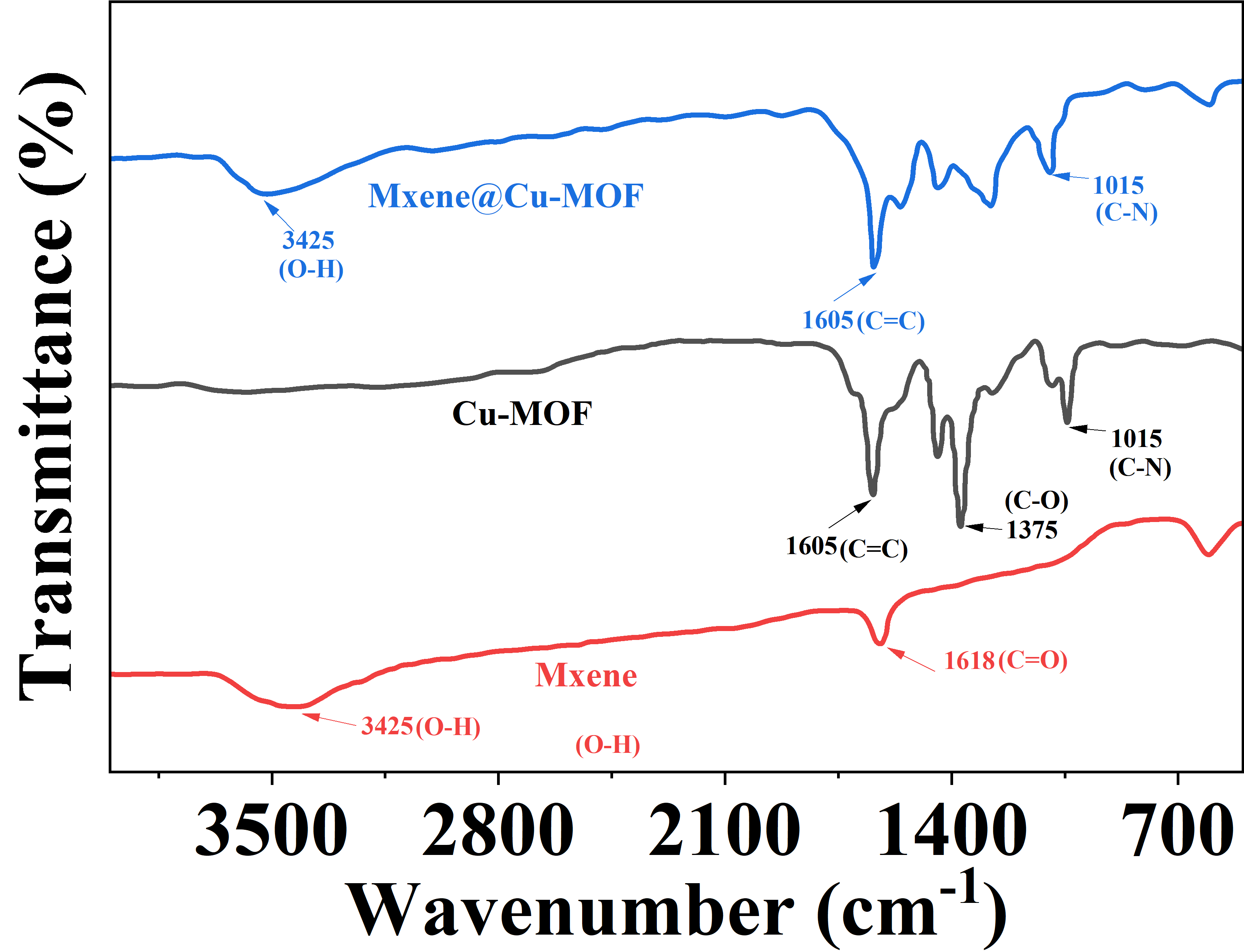


**Figure S4. FTIR spectra of MXene, Cu-MOF, and MXene@Cu-MOF.** FTIR spectra (ATR, 4000–400 cm^-1^) comparing MXene (surface –OH/–O terminations), Cu-MOF (ligand-related bands), and the MXene@Cu-MOF heterostructure. Characteristic peaks are labeled in the panels; the heterostructure exhibits combined signatures with interfacial shifts/attenuation.

**
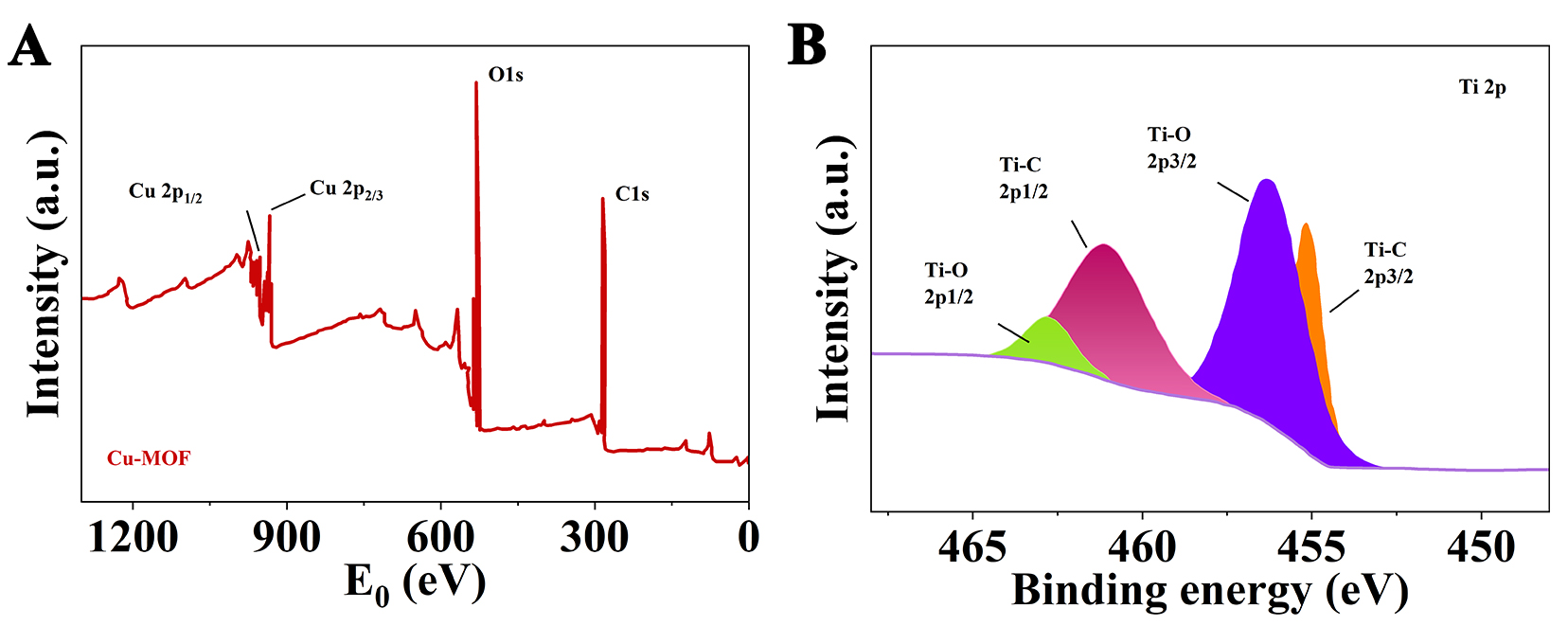
**

**Figure S5. XPS characterization.** (A) XPS survey (wide-scan) spectrum of Cu-MOF. (B) High-resolution XPS spectra of MXene (e.g., Ti-O 2p, and Ti-C 2p; peaks and fittings as indicated).


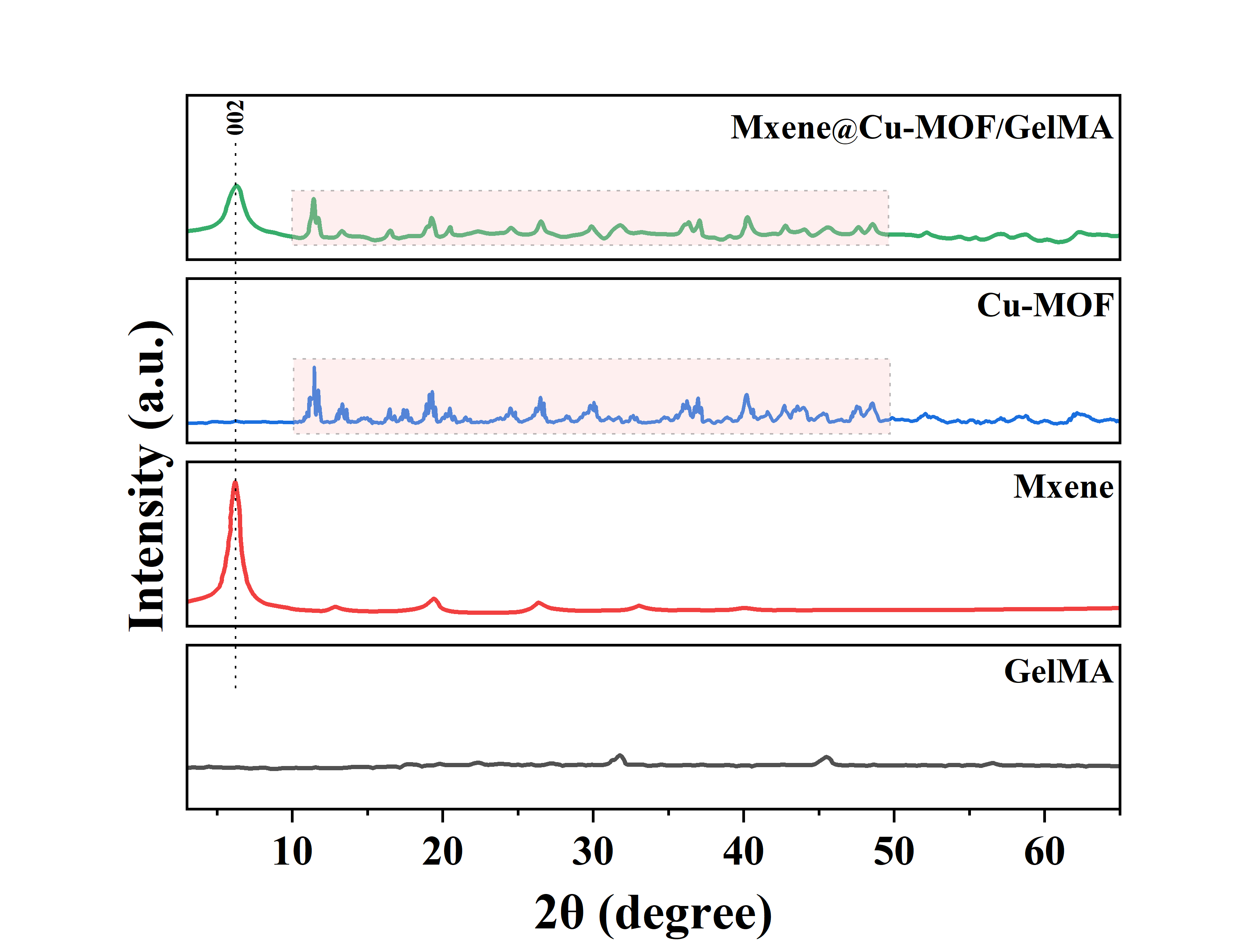


**Figure S6.** XRD profiles of GelMA, MXene (Ti_3_C_2_T_x_), Cu-MOF, and the MXene@Cu-MOF/GelMA platform.

**
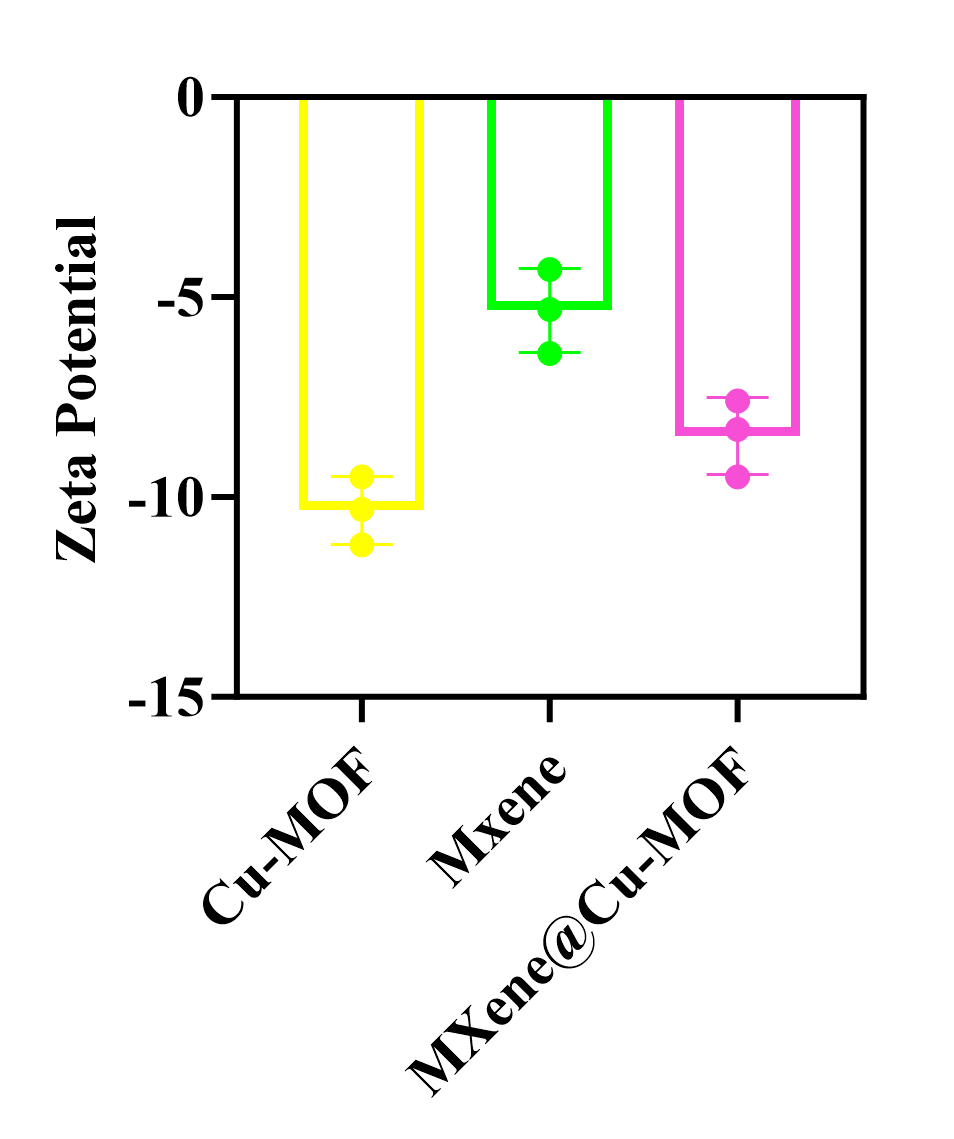
**

**Figure S7. ζ potential of Cu-MOF, MXene, and MXene@Cu-MOF.** Electrophoretic light-scattering measurements of ζ-potential distributions (histograms) with corresponding mean values (bar plots, mean ± SD; n = 3) for Cu-MOF, MXene (Ti_3_C_2_T_x_), and the Mxene@Cu-MOF heterostructure in aqueous electrolyte.

**
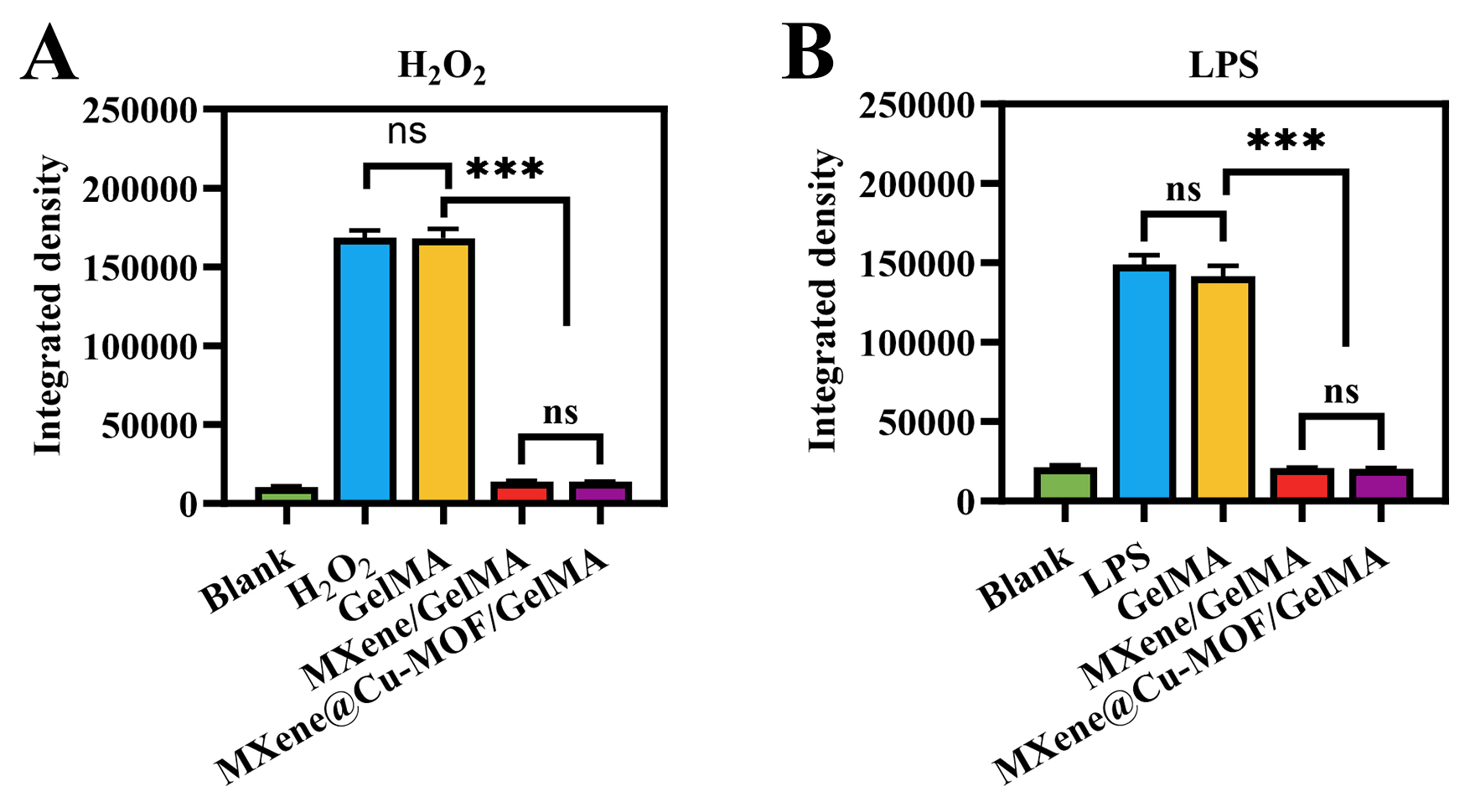
**

**Figure** **S8. Quantification of intracellular ROS in RAW264.7 macrophages.** (A) DCFH-DA–derived fluorescence intensity in cells treated with: PBS (blank), H_2_O_2_ alone, H_2_O_2_ + GelMA, H_2_O_2_ + MXene/GelMA, and H_2_O_2_ + MXene@Cu-MOF/GelMA.

(B) DCFH-DA fluorescence intensity in cells treated with: PBS (blank), LPS alone, LPS + GelMA, LPS + MXene/GelMA, and LPS + MXene@Cu-MOF/GelMA. Data are presented as mean ± SD (n = 3); ***P < 0.001; ns, not significant.

**
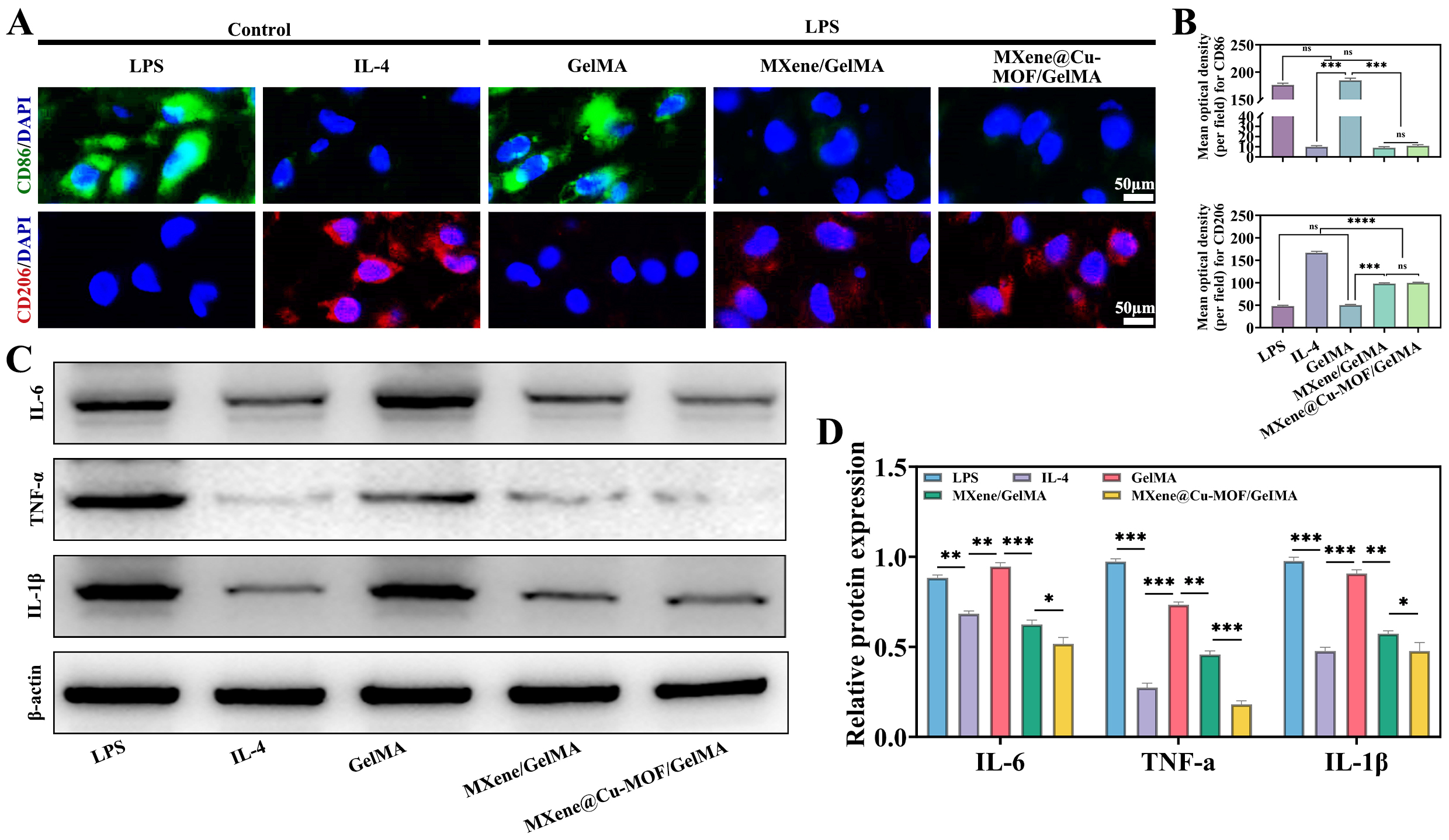
**

**Figure** **S9. Anti-inflammatory effects of the MXene@Cu-MOF/GelMA platform in LPS-prestimulated RAW264.7 macrophages.** (A,B) Representative immunofluorescence of M1 (CD86, green) and M2 (CD206, red) markers with corresponding quantification (normalized MFI or % positive cells, as indicated) in RAW264.7 cells treated with: LPS (positive control), IL-4 (negative/M2 control), LPS + GelMA, LPS + MXene/GelMA, and LPS + MXene@Cu-MOF/GelMA. Nuclei were counterstained with DAPI. (C,D) Western blot analysis of iNOS, IL-6, TNF-α, and IL-1β, with densitometric quantification normalized to the loading control. Data are presented as mean ± SD (n = 3); ***P < 0.001; ****P < 0.0001; ns, not significant.

**
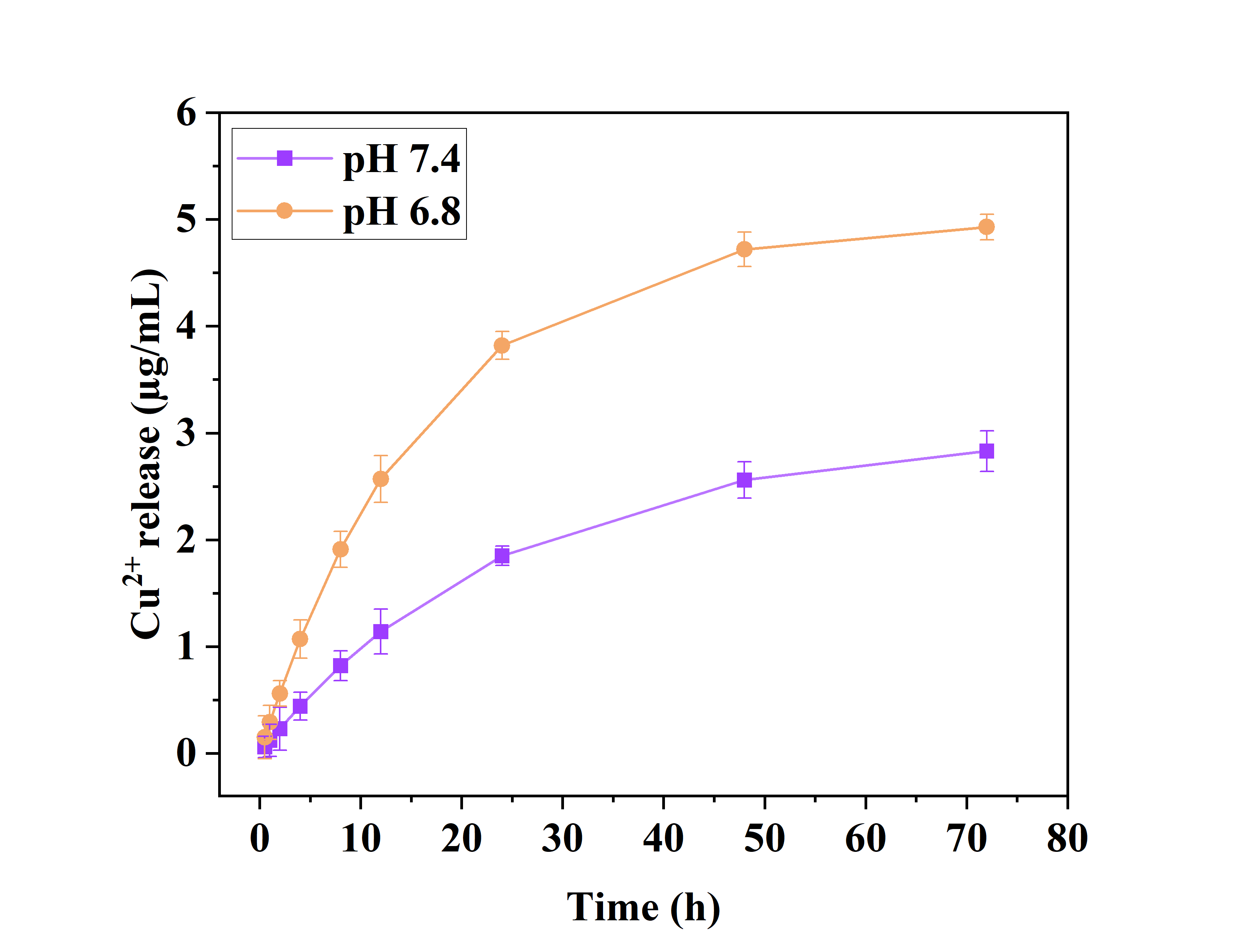
**

**Figure S10**. Cumulative Cu^2+^ release profiles from MXene@Cu-MOF/GelMA hydrogels under physiological (pH 7.4) and pathological (pH 6.8) conditions (PBS, 37℃). Data presented as mean ± SD (n = 3).

**
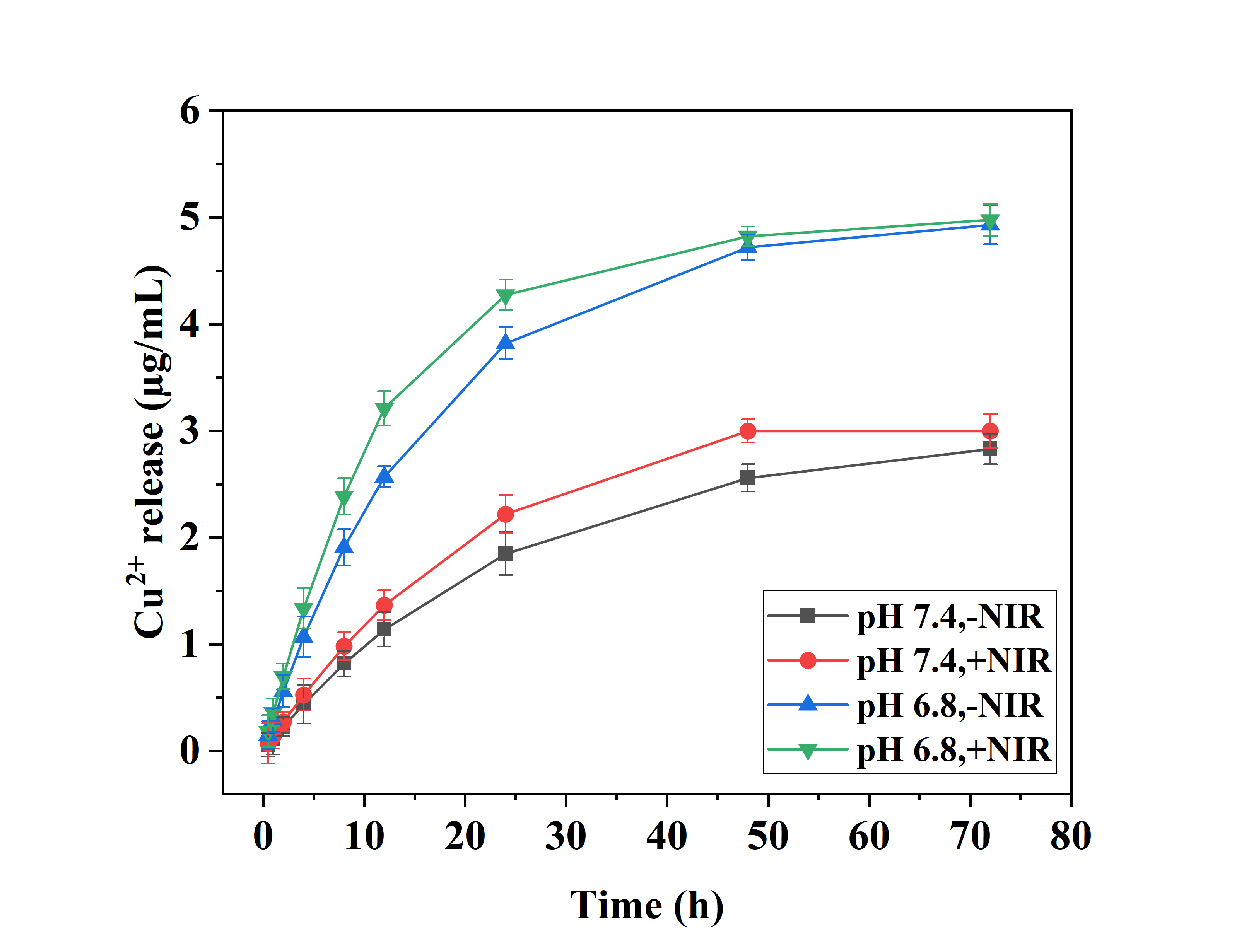
**

**Figure S11**. Cumulative Cu^2+^ release profiles from MXene@Cu-MOF/GelMA hydrogels under physiological (pH 7.4) and pathological (pH 6.8) conditions with or without 808 nm NIR irradiation. Data presented as mean ± SD (n = 3).

**
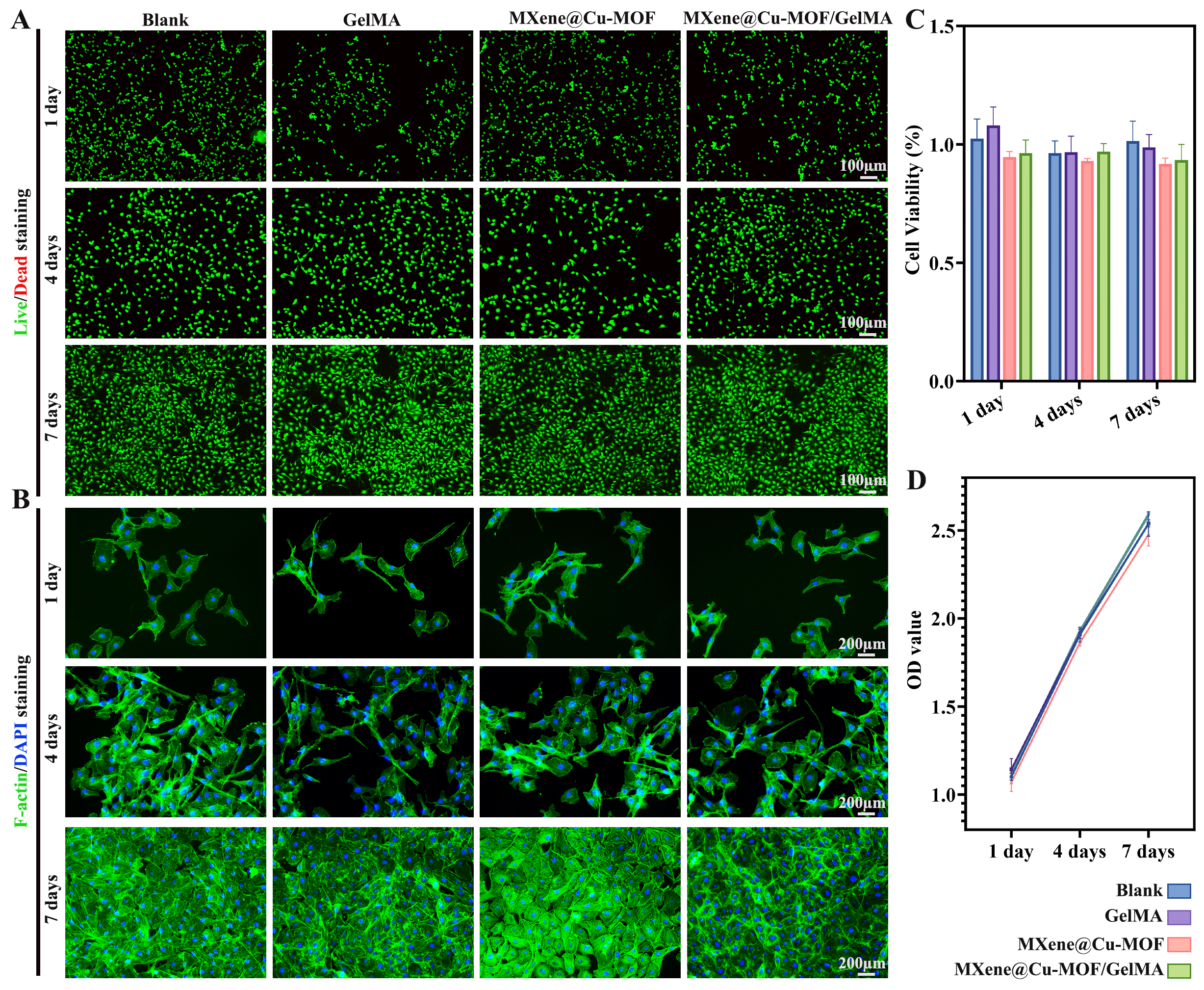
**

**Figure S12. *In vitro* cytocompatibility of MXene@Cu-MOF/GelMA hydrogels with primary chondrocytes.** (A) Live/Dead staining of chondrocytes cultured with Blank, GelMA, MXene@Cu-MOF, and MXene@Cu-MOF/GelMA for 1, 4, and 7 days. Green: live cells; red: dead cells. (B) F-actin/DAPI staining of chondrocytes on different substrates at 1, 4, and 7 days. (C) Cell viability at days 1, 4, and 7 as determined by the CCK-8 assay. (D) Time-dependent changes in OD values at days 1, 4, and 7 as measured by the CCK-8 assay. Data are presented as mean ± SD (n = 3).

**
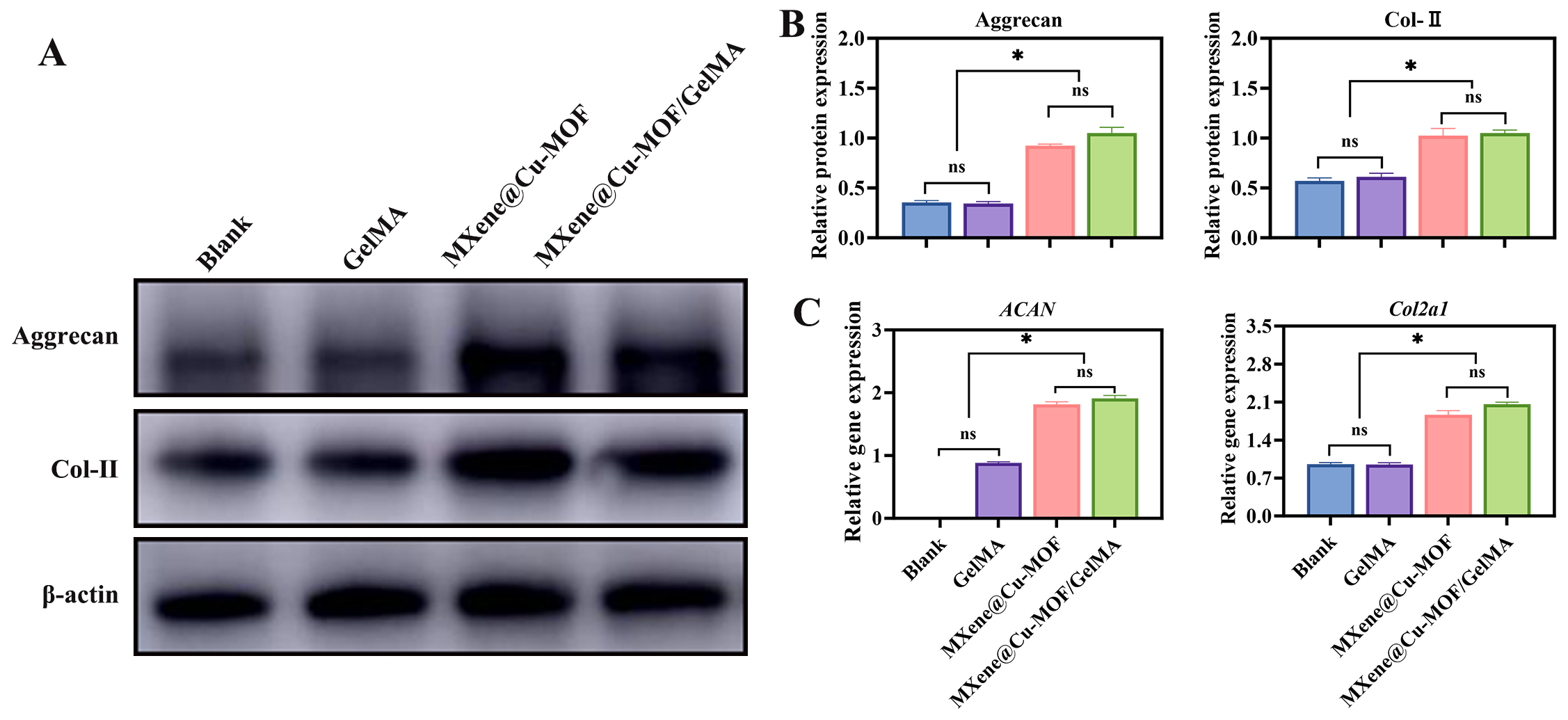
**

**Figure S13. Expression of chondrogenic markers in the repaired tracheal tissues.** (A) Western blot bands of aggrecan and Col-II proteins in chondrocytes cultured under Blank, GelMA, MXene@Cu-MOF, and MXene@Cu-MOF/GelMA conditions. (B) Quantitative analysis of aggrecan and Col-II proteins expression based on image A. (C) Relative mRNA expression levels of chondrogenic genes (*ACAN* and *Col2a1*). Data are presented as mean ± SD (n = 3); *P < 0.05; ns, not significant.

**
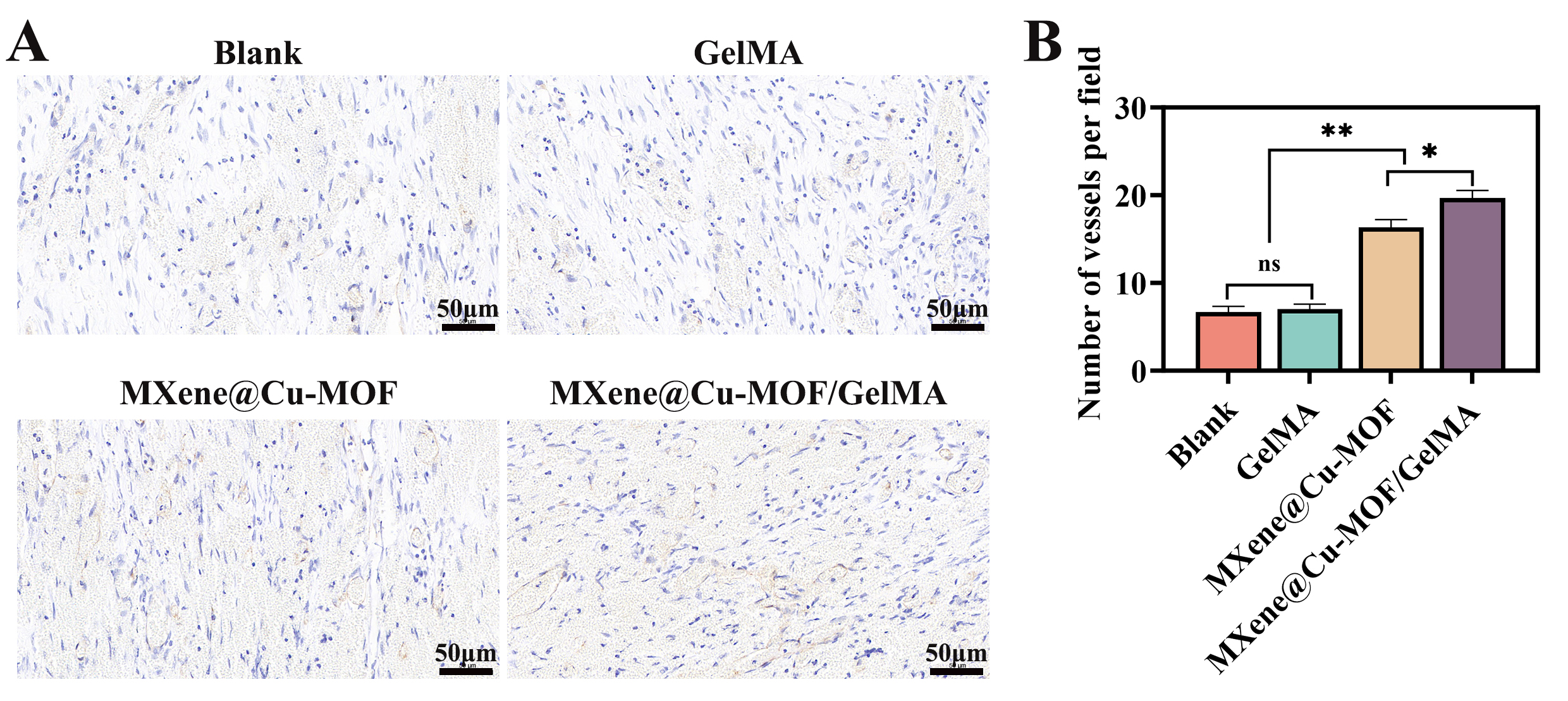
**

**Figure** **S14. *In vivo* neovascularization.** (A) Representative CD31 immunohistochemical staining of tissues surrounding the implantation sites in the Blank, GelMA, MXene@Cu-MOF, and MXene@Cu-MOF/GelMA groups. (B) Quantification of CD31^+^ microvessels (number of vessels per field) for each group. Data are presented as mean ± SD (n = 3); *P < 0.05; **P < 0.01; ns, not significant.

**
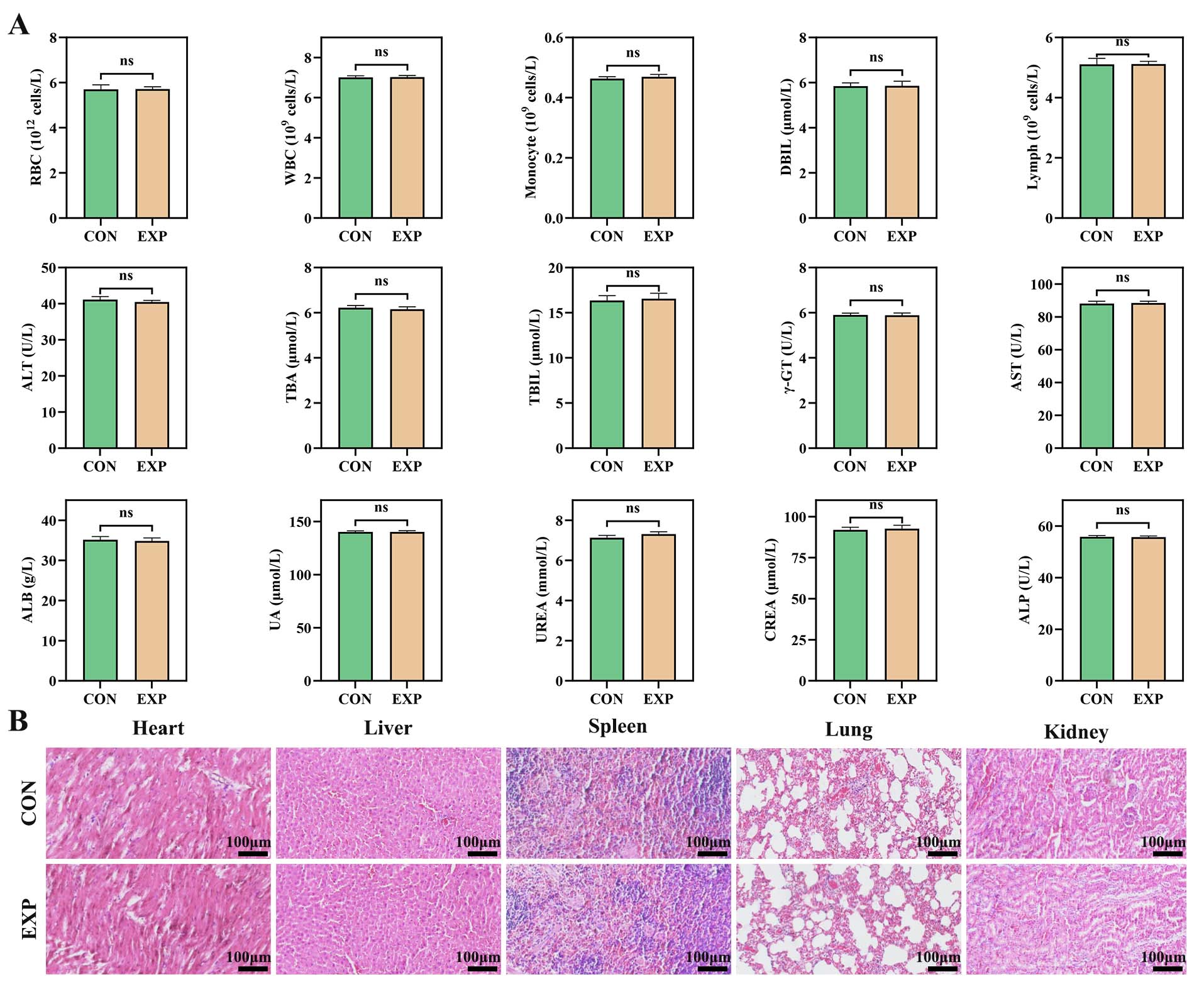
**

**Figure S15**. **Evaluation of systemic toxicity of MXene@Cu-MOF/GelMA hydrogel in an orthotopic rabbit tracheal defect model.** (A) Hematological and serum biochemical analyses of control (CON) and hydrogel-treated (EXP) rabbits at 4 weeks post-implantation, including complete blood count parameters (RBC, WBC, monocytes, lymphocytes) and liver/kidney function markers (DBIL, ALT, TBA, TBIL, γ-GT, AST, ALB, UA, UREA, CREA, ALP). (B) Representative H&E-stained sections of major organs (heart, liver, spleen, lung and kidney) from CON and EXP groups at 4 weeks post-implantation. Data are presented as mean ± SD (n = 3); ns, not significant.

**
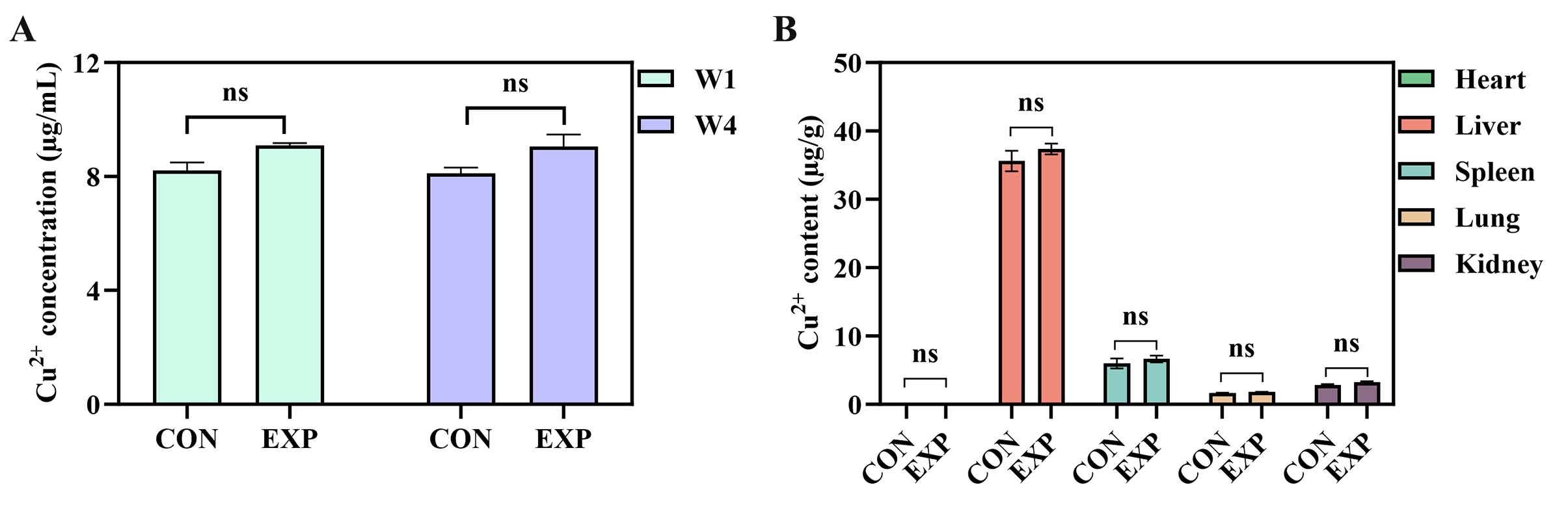
**

**Figure S16. Cu biodistribution and clearance in rabbits after MXene@Cu-MOF/GelMA implantation.** (A) Serum Cu^2+^ concentration in control (CON) and hydrogel-treated (EXP) rabbits at 1 week (W1) and 4 weeks (W4) post-operation. (B) Cu^2+^ content in major organs (heart, liver, spleen, lung, kidney) of CON and EXP groups at W4, expressed as µg/g wet tissue. Data are presented as mean ± SD (n = 3); ns, not significant.

**Table S1.** **Primers used in the qPCR**

| **Genes** | **Forward (5’–3’)** | **Reverse (5’–3’)** |
| --- | --- | --- |
| ***eNOS*** | TGTCCAACATGCTGCTGGAAATTG | AGGAGGTCTTCTTCCTGGTGATGCC |
| ***HIF-1α*** | AGTTCCGCAAGCCCTGAAAGC | GCAGTGGTAGTGGTGGCATTAGC |
| ***VEGF*** | TATGCGGATCAAACCTCACCA | GAAGAAAGTGGTGCCATGGATAG |
| ***FGF2*** | CAATTCCCATGTGCTGTGAC | ACCTTGACCTCTCAGCCTCA |
| ***GAPDH*** | AGGTCGGTGTGAACGGATTTG | TGTAGACCATGTAGTTGAGGTCA |
| ***Col2a1*** | GTGTCAGGGCCAGGATGT | TCCCAGTGTCACAGACACAGAT |
| ***ACAN*** | GTGCCTATCAGGACAAGGTCT | GATGCCTTTCACCACGACTTC |

**Table S2. Element composition of Cu-MOF, MXene, and MXene@Cu-MOF**

| **Groups** | **Chemical elements** | **Weight percentage (%)** | **Atom percentage (%)** |
| --- | --- | --- | --- |
| **Cu-MOF** | C | 40.43 | 57.14 |
|  | N | 5.89 | 7.14 |
|  | O | 26.93 | 28.57 |
|  | Cu | 26.74 | 7.14 |
|  | Total content | 99.99 |  |
| **MXene** | C | 11.89 | 28.57 |
|  | O | 9.50 | 17.14 |
|  | F | 7.52 | 11.43 |
|  | Ti | 71.08 | 42.86 |
|  | Total content | 99.99 |  |
| **MXene@Cu-MOF** | C | 20.45 | 40.61 |
|  | N | 1.77 | 3.01 |
|  | O | 14.73 | 21.96 |
|  | Ti | 49.76 | 24.79 |
|  | F | 5.27 | 6.61 |
|  | Cu | 8.02 | 3.01 |
|  | Total content | 100 |  |
